# Efficacy and Safety of Bevacizumab Biosimilars Compared With Reference Biologics in Advanced Non-small Cell Lung Cancer or Metastatic Colorectal Cancer Patients: A Network Meta-Analysis

**DOI:** 10.3389/fphar.2022.880090

**Published:** 2022-07-05

**Authors:** Xinyi Xu, Shengzhao Zhang, Ting Xu, Mei Zhan, Chen Chen, Chenyu Zhang

**Affiliations:** ^1^ Department of Pharmacy, West China Hospital, Sichuan University, Chengdu, China; ^2^ West China School of Pharmacy, Sichuan Unversity, Chengdu, China; ^3^ Deparment of Pharmacy, Karamay Centeral Hospital, Karamay, China

**Keywords:** biosimilars, bevacizumab, non-small cell lung cancer, meta-analysis, reference biologics, metastatic colorectal cancer

## Abstract

**Background:** Bevacizumab biosimilars are slowly making their way into cancer treatment, but the data on their efficacy and safety in cancer patients are still poor. We systematically summarized the current evidence for the efficacy and safety of bevacizumab biosimilars in patients with advanced non-small cell lung cancer (NSCLC) or metastatic colorectal cancer (CRC).

**Methods:** This review searched CNKI, VIP, PubMed, Medline (Ovid), Embase, and Cochrane Library (Ovid) for randomized controlled trials of bevacizumab biosimilars treated in adults with advanced NSCLC or metastatic CRC. A pairwise meta-analysis and a Bayesian network meta-analysis based on the random-effect model were performed to summarize the evidence. We rated the certainty of evidence according to the Grading of Recommendations Assessment, Development, and Evaluation framework.

**Results:** Ten eligible trials with a total of 5526 patients were included. Seven trials (n = 4581) were for the NSCLC population, while three trials (n = 945) were for patients with CRC. According to the pairwise meta-analysis, the efficacy (objective response rate: risk ratio (RR) 0.98 [0.92–1.04], *p* = 0.45; progression-free survival: hazard ratio (HR) 1.01 [0.92–1.10], *p* = 0.85; and overall survival: HR 1.06 [0.94–1.19], *p* = 0.35) and safety (incidence of grade 3–5 adverse events: odds ratio (OR) 1.03 [0.91–1.16], *p* = 0.65) of bevacizumab biosimilars performed no significant difference with reference biologics in patients with NSCLC as well as metastatic CRC patients (objective response rate: RR 0.97 [0.87–1.09], *p* = 0.60; overall survival: HR 0.94 [0.70–1.25], *p* = 0.66; incidence of grade 3–5 adverse events: OR 0.78 [0.59–1.02], *p* = 0.73). Network estimates displayed 7 types of bevacizumab biosimilars in the medication regime of NSCLC patients who had no significant difference among each other in terms of efficacy and safety. The certainty of the evidence was assessed as low to moderate. Three types of biosimilars were found to be clinically equivalent to each other in the patients with CRC, which were evaluated with very low to moderate certainty.

**Conclusion:** In patients with advanced NSCLC or metastatic CRC, the efficacy and safety of bevacizumab biosimilars were found to be comparable with those of reference biologics and each other.

## 1 Introduction

According to the latest GLOBOCAN estimates, lung cancer and colorectal cancer (CRC) are ranked as the second and third most common cancers in 2020, respectively, and are ranked as the first and second leading causes of cancer deaths worldwide, respectively (Siegel et al., 2020; Sung et al., 2021). Bevacizumab combined with platinum-based chemotherapy was approved for the first-line treatment of a variety of malignancies, including non-small cell lung cancer (NSCLC) and CRC, in the United States, European Union, and China (EMA, 2018; Garcia et al., 2020; Genentech Inc., 2020). Bevacizumab is a recombinant humanized monoclonal antibody that binds and suppresses the biological activity of vascular endothelial growth factor (VEGF) by preventing its interaction with endothelial cell surface receptors (Kim et al., 1993).

As patents of a lot of biologics had expired, biosimilars, in particular bevacizumab, became a hot spot for companies and researchers (Safdar et al., 2021). Biosimilars refer to a biologic that is highly similar to the reference biologics with no clinically meaningful differences in purity, safety, and efficacy from the reference biologics (FDA, 2015). At present, the availability of many biosimilars in cancer is gradually rising (Kaida-Yip et al., 2018). The emergence of various biosimilars not only brings cost savings for patients but also emphasizes the necessity of patient access to anticancer therapies and the sustainability of cancer care (Abraham et al., 2014; Minion et al., 2015; Camacho, 2017; Simoens, 2021). ABP 215 (Mvasi^TM^) is the first approved bevacizumab biosimilar for the first-line treatment of patients with NSCLC by the Food and Drug Administration (FDA). Until April 2022, the FDA has approved three bevacizumab biosimilars, the National Medical Products Administration (NMPA) in China has approved eight bevacizumab biosimilars, and the European Medicines Agency (EMA) has approved ten bevacizumab biosimilars (EMA, 2022; FDA, 2022; NMPA, 2022). Thus far, equivalency studies have demonstrated that bevacizumab biosimilars were comparable with their reference biologics in the population of either NSCLC or CRC; nevertheless, the comparisons among different biosimilars were never made (Jichun Yang et al., 2019). This lack of evidence still makes it difficult for clinicians and payers to make informed judgments when faced with a variety of biosimilars.

As a result, we thoroughly analyzed the current evidence on the efficacy and safety of bevacizumab biosimilars in patients with NSCLC or CRC when compared with each other and reference biologics.

## 2 Materials and Methods

This study was mainly carried out using the guidelines of the Preferred Reporting Items for Systematic Reviews and Network Meta-analyses checklist (Hutton et al., 2015). This network meta-analysis (NMA) was registered on the International Prospective Register of Systematic Review (PROSPERO, CRD42022301478).

### 2.1 Literature Search

A comprehensive literature search was performed via the databases, including PubMed, Medline (Ovid), Embase, Cochrane Library (Ovid), CNKI (China National Knowledge Infrastructure), Wanfang, and VIP, to confirm all relevant studies published within the time range from database creation to September 23, 2021. The search terms mainly included “Bevacizumab,” “Biosimilar pharmaceuticals,” and “Avastin” (Supplementary Appendix S3).

### 2.2 Eligibility Criteria

The specific inclusion criteria were as follows: 1) Patients: ① histologically or cytologically diagnosed stage IIIB–IV firstly or recurrent CRC or nonsquamous NSCLC; ② a baseline Eastern Cooperative Oncology Group performance status of two or less; ③ had to have adequate bone marrow, hepatic, and renal function; ④ had at least one measurable lesion per Response Evaluation Criteria in Solid Tumors version 1.1 (RECIST 1.1 version); ⑤ a life expectancy ≥ 3 months; and ⑥ aged 18–75 years. 2) Intervention: bevacizumab biosimilars combined chemotherapy. 3) Comparison: bevacizumab reference biologics. 4) Outcomes: at least one of the following clinical outcome measures was reported: objective response rate (ORR), progression-free survival (PFS), overall survival (OS), and the incidence rate of grade 3–5 adverse events (AEs). 5) Study: random controlled trials (RCTs) in Chinese and English languages.

Patients with known central nervous system metastases (treated and stable brain metastases are allowed) or planned major surgery during the treatment phase were excluded. If patients had anaplastic lymphoma kinase (ALK) gene rearrangement or a history of allergic reactions to chemical or biological structures similar to bevacizumab, oxaliplatin and irinotecan (IRI), fluorouracil (5-FU), and/or leucovorin (LV), they were excluded. Populations with received metastatic or recurrent NSCLC first-line systemic neoadjuvant/adjuvant chemotherapy, targeted VEGF receptor or epidermal growth factor receptor signaling pathway, or immune therapy within ≤12 months before randomization, or recurrence within 6 months after adjuvant treatment were excluded. Importantly, adults with a diagnosis of cell lung cancer or a mixture of small cell lung cancer and NSCLC were excluded. Adults with active bleeding, clinically significant cardiovascular disease (unstable angina, myocardial infarction, or congestive heart failure), severe nonunion wounds, ulcers, fractures, or proteinuria or participants with uncontrolled hypertension or systolic blood pressure >140 mmHg or diastolic blood pressure >90 mmHg, diabetes, infection, or epilepsy were excluded. Pregnant or breastfeeding women were not included.

### 2.3 Study Selection and Data Extraction

Two members (XX and SZ) comprehensively screened articles from databases based on the eligibility criteria independently. Data extractions were also independently finished by the two reviewers above. Any discrepancies were resolved via discussion or through third-party adjudication (TX).

### 2.4 Risk of Bias

Two members of our team (XX and SZ) selected the Cochrane bias risk tool (RevMan software version 5.4) to independently assess the risk of bias of all included RCTs. Any discrepancies were resolved by discussion or by the third member (TX) (Higgins and Green, 2011). The results of publication bias were mainly presented with funnel plots.

### 2.5 Treatment Nodes

In this analysis, we classified the treatment nodes by drugs. The network plots were conducted using the *multinma* package in R (version 4.0.5).

### 2.6 Statistical Analysis

We conducted a pairwise meta-analysis to assess the efficacy and safety of biosimilars and reference biologics. We used hazard ratio (HR) and 95% credible interval (CI) as effect size measures to assess PFS and OS. An odds ratio (OR) with 95% CI was used to measure the incidence of grade 3–5 AEs, and a risk ratio (RR) with 95% CI was used to measure ORR. Heterogeneity among studies was assessed using the inconsistency test (*I* ^*2*^). In particular, if *I* ^*2*^ was greater than 50%, corresponding to a high risk of heterogeneity, then the meta-analysis was calculated using the random effect–based model; otherwise, the meta-analysis was carried out using the fixed effect-based model (Borenstein et al., 2010). Pairwise meta-analysis was conducted using the *meta* package in R software.

The NMA was conducted under the utilization of the random-effects model and consistency model in the Bayesian framework for different outcomes of different targeted patients (Röver, 2017). Four Markov chains with 32,000 iterations after an initial burn-in of 8,000 both with a thinning of one were utilized for the analysis of the outcomes above (Greco et al., 2016). In this NMA, we ranked treatments based on the surface under the cumulative ranking curve (SUCRA) values calculated from Bayesian analysis (Salanti et al., 2011). Consistency and transitivity, which were two critical assumptions of NMA, were evaluated (Cipriani et al., 2013). Transitivity was assessed using descriptive statistics from studies and population baselines (Cipriani et al., 2013). Concerning another critical assumption, consistency referred to direct and indirect estimates that were statistically similar (Cipriani et al., 2013). Node splitting methods were used to compute model inconsistencies, where direct and indirect evidence were compared at a node (particular comparison) separately. A significant difference was considered to exist if the *p*-value was less than 0.05. NMA was conducted using the *gemtc* package in R software.

### 2.7 Certainty of Evidence

GRADE (Grading of Recommendations Assessment, Development, and Evaluation) framework was used for pairwise meta-analysis and NMA was used to rate the certainty of the evidence (Guyatt et al., 2008; Puhan et al., 2014; Brignardello-Petersen et al., 2018; Brignardello-Petersen et al., 2019). The two members of our team (XX and SZ) were evaluated on the basis of the above, and any ambiguity was discussed and resolved.

## 3 Results

### 3.1 Systematic Review and Characteristics of Included Patients

**FIGURE 1:**
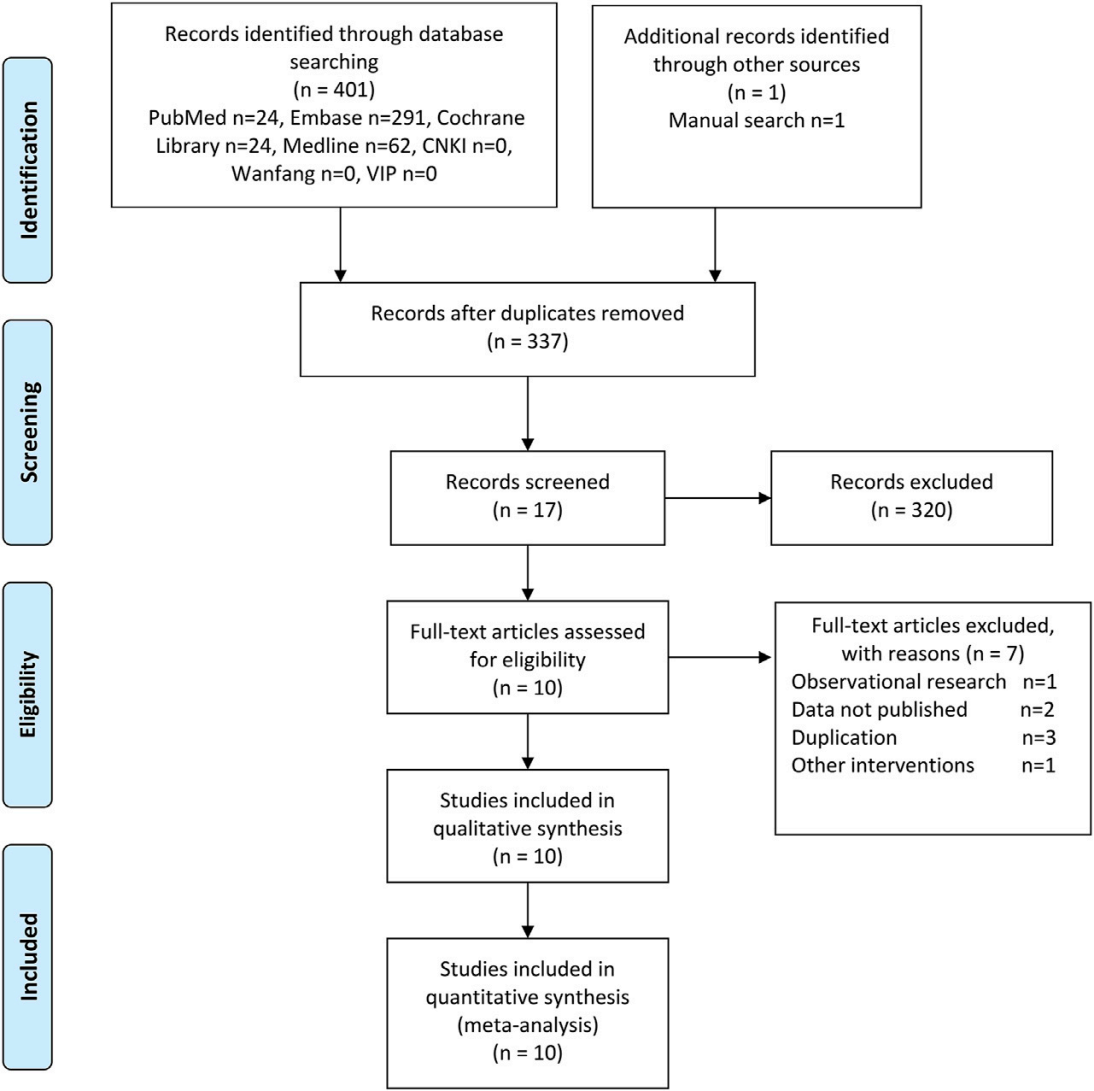
PRISMA flow diagram.

A total of 402 records from the above databases were identified, 17 reports in the full text were reviewed, and 10 RCTs with 5472 patients were deemed eligible for the criteria above (Figure 1) (Romera et al., 2018; Reinmuth et al., 2019; Thatcher et al., 2019; Yunpeng Yang et al., 2019; Reck et al., 2020; Rezvani et al., 2020; Qin et al., 2021; Shi et al., 2021; Syrigos et al., 2021; Trukhin et al., 2021). The baseline characteristics of these trials were summarized in Table 1.

**TABLE1:** Baseline characteristics of RCTs included.

Study	Biosimilars	Disease	Nct	Sample Size	Mean Age	Male (%)	Treatment Regimes		Outcomes	Approval
				B/R	B/R		B Group	R Group		
Reinmuth2019	PF-06439535 (Zirabev^TM^)	NSCLC	NCT02364999	358/361	62.0/61.0	65.0	PF-6439535: 15 mg/kg paclitaxel: 200 mg/m^2^ carboplatin: AUC 6	bevacizumab: 15 mg/kg paclitaxel: 200 mg/m^2^ carboplatin: AUC 6	ORR PFS OS g3-5 A E	FDA, EMA
Thatcher 2019	ABP 215 (Mvasi^TM^)	NSCLC	NCT01966003	328/314	61.6/61.6	59.8	ABP 215: 15 mg/kg paclitaxel: 200 mg/m^2^ carboplatin: AUC 6	bevacizumab: 15 mg/kg paclitaxel: 200 mg/m^2^ carboplatin: AUC 6	ORR g3-5 A E	FDA, EMA
Reck2020	SB8 (Aybintio^TM^)	NSCLC	NCT02754882	379/384	60.2/60.0	66.6	SB8: 15 mg/kg paclitaxel: 200 mg/m^2^ carboplatin: AUC 6	bevacizumab: 15 mg/kg paclitaxel: 200 mg/m^2^ carboplatin: AUC 6	ORR PFS OS g3-5 A E	EMA
Syrigos 2021	FKB238	NSCLC	NCT02810457	364/367	60.8/61.1	66.1	FKB238: 15 mg/kg paclitaxel: 200 mg/m^2^ carboplatin: AUC 6	bevacizumab: 15 mg/kg paclitaxel: 200 mg/m^2^ carboplatin: AUC 6	ORR PFS OS g3-5 A E	
Trukhin 2021	MB02 (Alymsys^TM^)	NSCLC	NCT03296163	315/312	61.0/61.0	61.1	MB02: 15 mg/kg paclitaxel: 200 mg/m^2^ carboplatin: AUC	bevacizumab: 15 mg/kg paclitaxel: 200 mg/m^2^ carboplatin: AUC 6	ORR PFS OS g3-5 A E	FDA, EMA
Yang2019	IBI305 (Byvasda^TM^)	NSCLC	NCT02954172	224/226	57.6/57.2	63.3	IBI305: 15 mg/kg paclitaxel: 175 mg/m^2^ carboplatin: AUC 6	bevacizumab: 15 mg/kg paclitaxel: 175 mg/m^2^ carboplatin: AUC 6	ORR g3-5AE	NMPA
Shi2021	LY01008 (Boyounuo^TM^)	NSCLC	NCT03533127	324/325	58.0/59.0	59.8	LY01008: 15 mg/kg paclitaxel: 175 mg/m^2^ carboplatin: AUC 6	bevacizumab: 15 mg/kg paclitaxel: 175 mg/m^2^ carboplatin: AUC 6	ORR g3-5AE	NMPA
Rezvani 2020	BE1040V	CRC	NCT03288987	82/44	56.3/56.3	36.5	BE1040V: 5 mg/kg* FOLFIRI-3	bevacizumab: 5 mg/kg* FOLFIRI-3	ORR OS g3-5AE	
Romera 2018	BEVZ92 (Alymsys^TM^)	CRC	NCT02069704	71/71	56.3/56.7	55.7	BEVZ92: 5 mg/kg* FOLFIRI or FOLFOX	bevacizumab: 5 mg/kg* FOLFIRI or FOLFOX	ORR g3-5AE	FDA, EMA
Qin2021	HLX04 (Hanbeitai^TM^)	CRC	NCT03511963	340/337	56.7/57.4	59.7	(1) HLX04: 7.5 mg/kg XELOX (2) HLX04: 5 mg/kg* mFOLFOX6	(1) HLX04: 7.5 mg/kg XELOX (2) HLX04: 5 mg/kg* mFOLFOX6	ORR OS g3-5AE	NMPA

FOLFIRI-3: ①IRI (oxaliplatin and irinotecan): 100 mg/m^2^ ivgtt. day 1, 100 mg/m^2^ ivgtt. day 3 of 14 days, ②5-FU: 2000 mg/m^2^ ivgtt. day 1 of 14 days, ③leucovorin: 400 mg/m^2^ iv. day 1 of 14 days; XELOX: ①oxaliplatin: 130 mg/m^2^ ivgtt. day 1 of 21 days, ②capecitabine: 1000 mg/m^2^ po., t.i.d., day 1–14 of 21 days; mFOLFOX6: ①oxaliplatin: 85 mg/m^2^ ivgtt. day 1 of 14 days, ②leucovorin: 400 mg/m^2^ iv. day 1 of 14 days, ③5-FU: 400 mg/m^2^ iv. day 1 followed by 2400 mg/m^2^ × 46 h continuous intravenous infusion of 14 days; FOLFIRI (fluorouracil, leucovorin, and irinotecan) or FOLFOX (fluorouracil, leucovorin, and oxaliplatin); g3-5 A E: Incidence rate of 3–5 grade adverse events; ORR: objective response rate; PFS: Progression-free survival; OS: overall survival; *: Carrying this symbol indicated that a cycle of the scheme was 14days, otherwise it was 21day; FDA: food and drug administration; EMA: european medicines agency; NMPA: national medical products administration.

Seven RCTs were on NSCLC, including a total of 4581 patients, with an average age of 57.2–62.0 years, and the proportion of male patients was approximately 59.8%–66.6%. For NSCLC, all patients received the same medication regimen: one of the bevacizumab biosimilars or reference biologics (15 mg/kg) along with carboplatin (AUC 6) and paclitaxel (175/200 mg/m^2^) once 3 weeks. Related biosimilars above mainly involved seven different types, including PF-06439535 (Zirabev^TM^) (Reinmuth et al., 2019), ABP 215 (Mvasi^TM^) (Thatcher et al., 2019), SB8 (Aybintio^TM^) (Reck et al., 2020), FKB238(Syrigos et al., 2021), MB02 (Alymsys^TM^) (Trukhin et al., 2021), IBI305 (Byvasda^TM^) (Yunpeng Yang et al., 2019), and LY01008 (Boyounuo^TM^) (Shi et al., 2021). At present, all biosimilars except FKB238 have been approved to market in the clinical environment.

A total of 945 patients with CRC were included in three RCTs with a mean age of 56.3–56.7 years, and the proportion of male patients was approximately 36.5%–56.7%. The medication regimens of CRC patients are not completely consistent, with the majority of patients receiving one of the bevacizumab biosimilars and reference biologics (5/7.5 mg/kg) in combination with either FOLFIRI (IRI, 5-FU, and LV), FOLFOX (fluorouracil, LV, and oxaliplatin), or XELOX (oxaliplatin and capecitabine). There were three biosimilars [BE1040V (Rezvani et al., 2020), BEVZ92 (Romera et al., 2018), and HLX04 (Qin et al., 2021)] in the treatment of CRC. In particular, BEVZ92 (Alymsys^TM^) and HLX04 (Hanbeitai^TM^) were approved for marketing.

### 3.2 Risk of Bias in Eligible Studies

The assessment summary outcomes for risk of bias about eligible RCTs are presented in Supplementary Figure S1. Specifically, all studies, except one with high risk in blinding of participants and outcome assessment, were revealed at low risk. The assessment of the quality of evidence for pairwise meta-analysis is summarized in Supplementary Table S1. We believed that there might be no apparent publication bias based on the symmetry of the funnel plot (Supplementary Figures S2 and S3).

### 3.3 Pairwise Meta-Analysis

#### 3.3.1 NSCLC Patients

**FIGURE 2:**
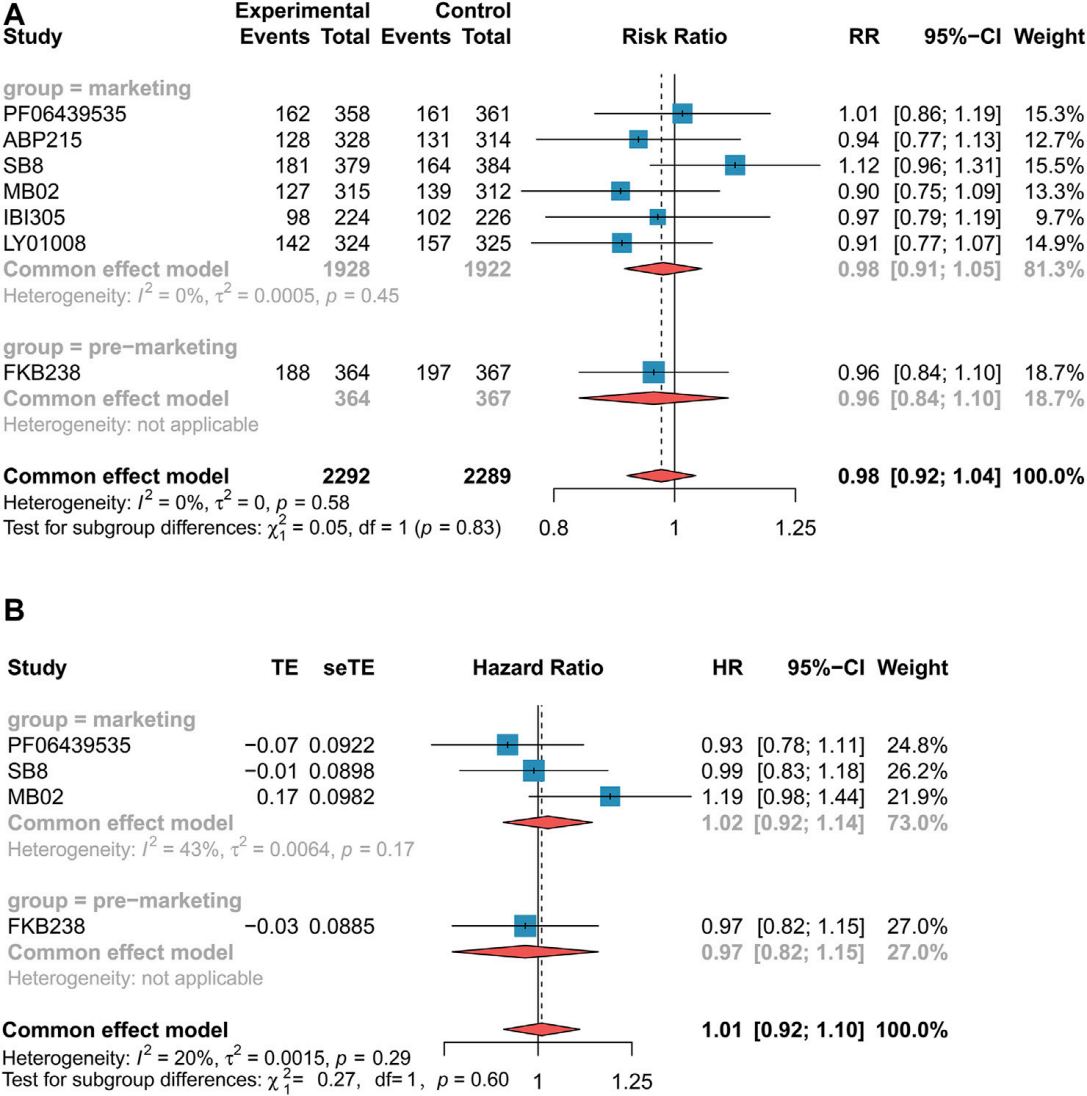
Forest plots of objective response rate and progression-free survival for non-small cell lung cancer patients. **(A)** Objective response rate. **(B)** Progression-free survival. These drugs were grouped by marketing. The marketing drug included PF-06439535, ABP 215, SB8, MB02, IBI305, and LY01008, while FKB238 belonged to the premarketing drug. Objective response rate was measured using risk ratio (RR), and progression-free survival was measured using hazard ratio (HR). If the RR value was higher than 1, it favored the biosimilar group; otherwise, it favored the reference biologics group. If the HR value was more than 1, it favored the biosimilar group; otherwise, it favored the reference biologics group. CI, confidence interval; RR, risk ratio; and HR, hazard ratio.

**FIGURE 3:**
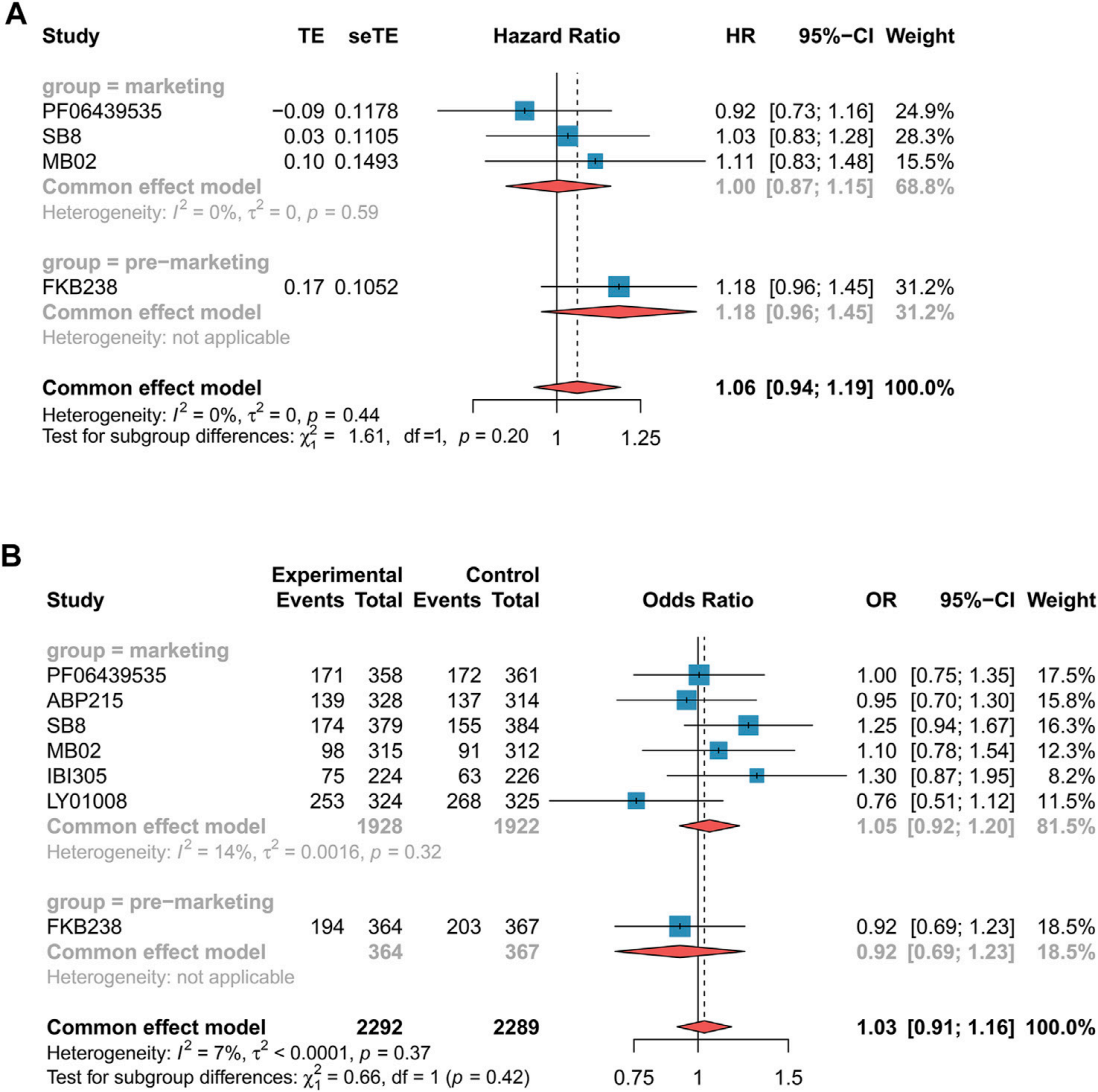
Forest plots of overall survival and incidence rate of grade 3–5 adverse events for non-small cell lung cancer patients. **(A)** Overall survival. **(B)** Incidence of grade 3–5 adverse events. These drugs were grouped by marketing. The marketing drug included PF-06439535, ABP 215, SB8, MB02, IBI305, and LY01008, while FKB238 belonged to the premarketing drug. Overall survival and incidence of grade 3–5 adverse events were measured using hazard ratio (HR) and odds ratio (OR), respectively. If the HR value was more than 1, it favored the biosimilar group; otherwise, it favored the reference group. If the OR value is higher than 1, it favored the reference biologics group; by contrast, it favored the biosimilars group. CI, confidence interval; OR, odds ratio; and HR, hazard ratio.

ORR and incidence rate of grade 3–5 AEs were evaluated in seven RCTs for NSCLC patients (Reinmuth et al., 2019; Thatcher et al., 2019; Yunpeng Yang et al., 2019; Reck et al., 2020; Shi et al., 2021; Syrigos et al., 2021; Trukhin et al., 2021), while the data of PFS and OS were available only by four RCTs (Reinmuth et al., 2019; Reck et al., 2020; Syrigos et al., 2021; Trukhin et al., 2021). The pooled results showed no significant differences in ORR (RR 0.98 [0.92–1.04], *p* = 0.45, *I* ^*2*^ = 0, low certainty, Figure 2A), PFS (HR 1.01 [0.92–1.10], *p* = 0.85, *I* ^*2*^ = 20%, moderate certainty, Figure 2B), or OS (HR 1.06 [0.94–1.19], *p* = 0.35, *I* ^*2*^ = 0, moderate certainty, Figure 3A) between biosimilars and reference biologics in NSCLC patients. As for the safe outcome, the incidence rate of grade 3–5 AEs was consistent with efficacy outcomes (OR 1.03 [0.91–1.16], *p* = 0.65, *I* ^*2*^ = 7%, moderate certainty, Figure 3B). For the subgroup receiving these biosimilars that had been approved for marketing, there was no significant difference in efficacy and safety from those receiving the reference biologics (ORR: RR 0.98 [0.91–1.05], *p* = 0.45; PFS: HR 1.02 [0.92–1.14], *p* = 0.85; OS: HR 1.00 [0.87–1.15], *p* = 0.35; incidence rate of grade 3–5 AEs: OR 1.05 [0.92–1.20], *p* = 0.65).

#### 3.3.2 CRC Patients

**FIGURE 4:**
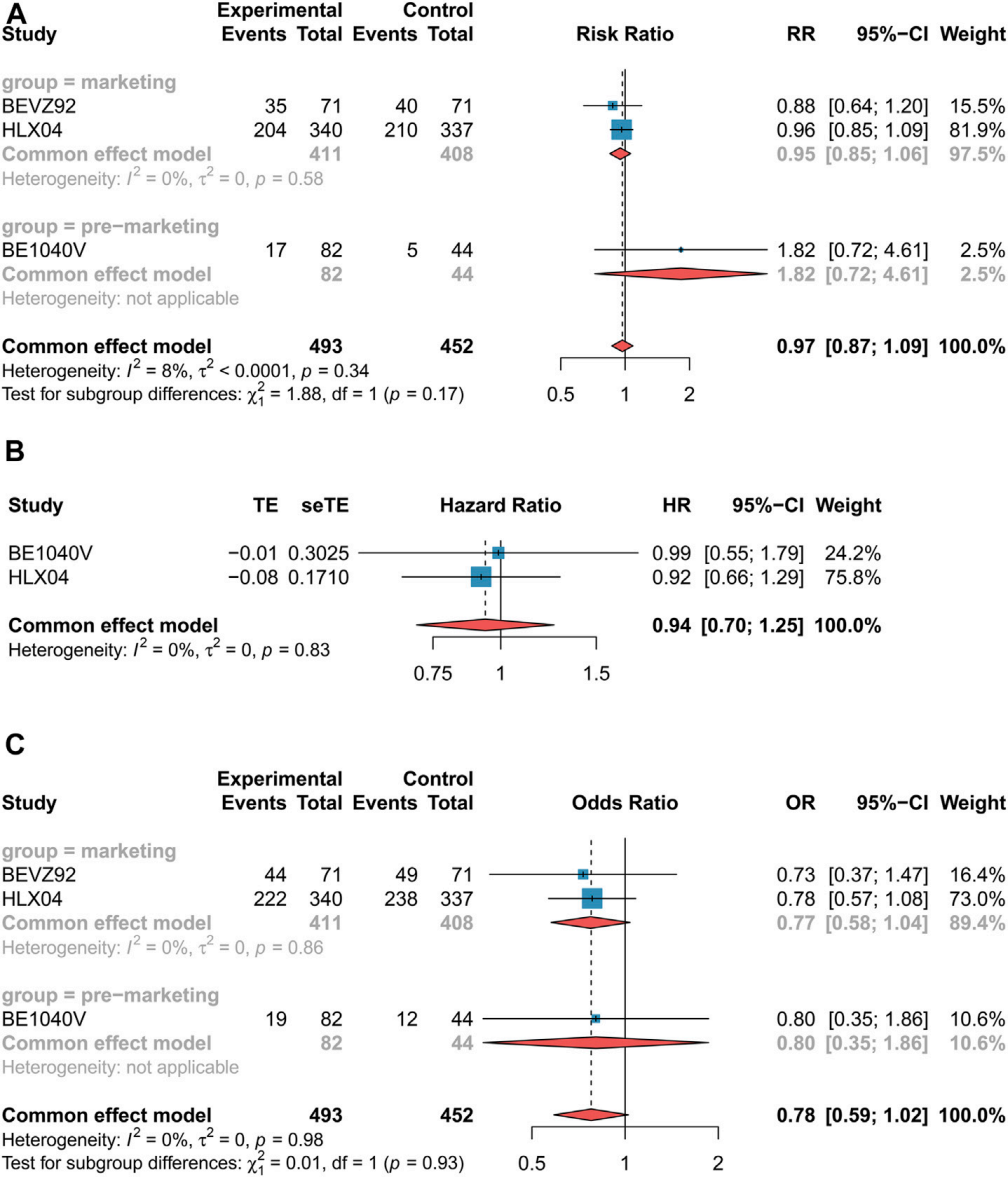
Forest plots of outcomes for colorectal cancer patients. **(A)** Objective response rate. **(B)** Overall survival. **(C)** Incidence of grade 3–5 adverse events. These drugs were grouped by marketing. The marketing drug included BEVA92 and HLX04, while BE1040V belonged to the premarketing drug. Objective response rate was measured using risk ratio (RR), overall survival was measured using hazard ratio (HR), and incidence of grade 3–5 adverse events were measured using odds ratio (OR). If the RR value was higher than 1, it favored the biosimilar group; otherwise, it favored the reference biologics group. If the HR value was more than 1, it favored the biosimilar group; otherwise, it favored the reference biologics group; If the OR value is higher than 1, it favored the reference biologics group; by contrast, it favored the biosimilars group. CI, confidence interval; OR, odds ratio; HR, hazard ratio; and RR, risk ratio.

We assessed ORR and incidence rate of grade 3–5 AEs in three RCTs (Romera et al., 2018; Rezvani et al., 2020; Qin et al., 2021). Nevertheless, the data of OS were reported only from two RCTs (Rezvani et al., 2020; Qin et al., 2021). The clinical efficacy (ORR: RR 0.97 [0.87–1.09], *p* = 0.60, *I* ^*2*^ = 8%, very low certainty, Figure 4A; OS: HR 0.94 [0.70–1.25], *p* = 0.66, *I* ^*2*^ = 0, low certainty, Figure 4B) and safety (AEs: OR 0.78 [0.59–1.02], *p* = 0.73, *I* ^*2*^ = 0%, low certainty, Figure 4C) of bevacizumab biosimilars were found to be comparable with reference biologics in CRC patients. In terms of marketing biosimilars, the subgroup that received these drugs was equivalent to the population of reference biologics (ORR: RR 0.95 [0.85–1.06], *p* = 0.60; OS: HR 0.94 [0.70–1.25], *p* = 0.66; incidence rate of grade 3–5 AEs: OR 0.77 [0.58–1.04], *p* = 0.73).

### 3.4 Network Meta-Analysis

#### 3.4.1 Results of Network Meta-Analysis

**FIGURE 5:**
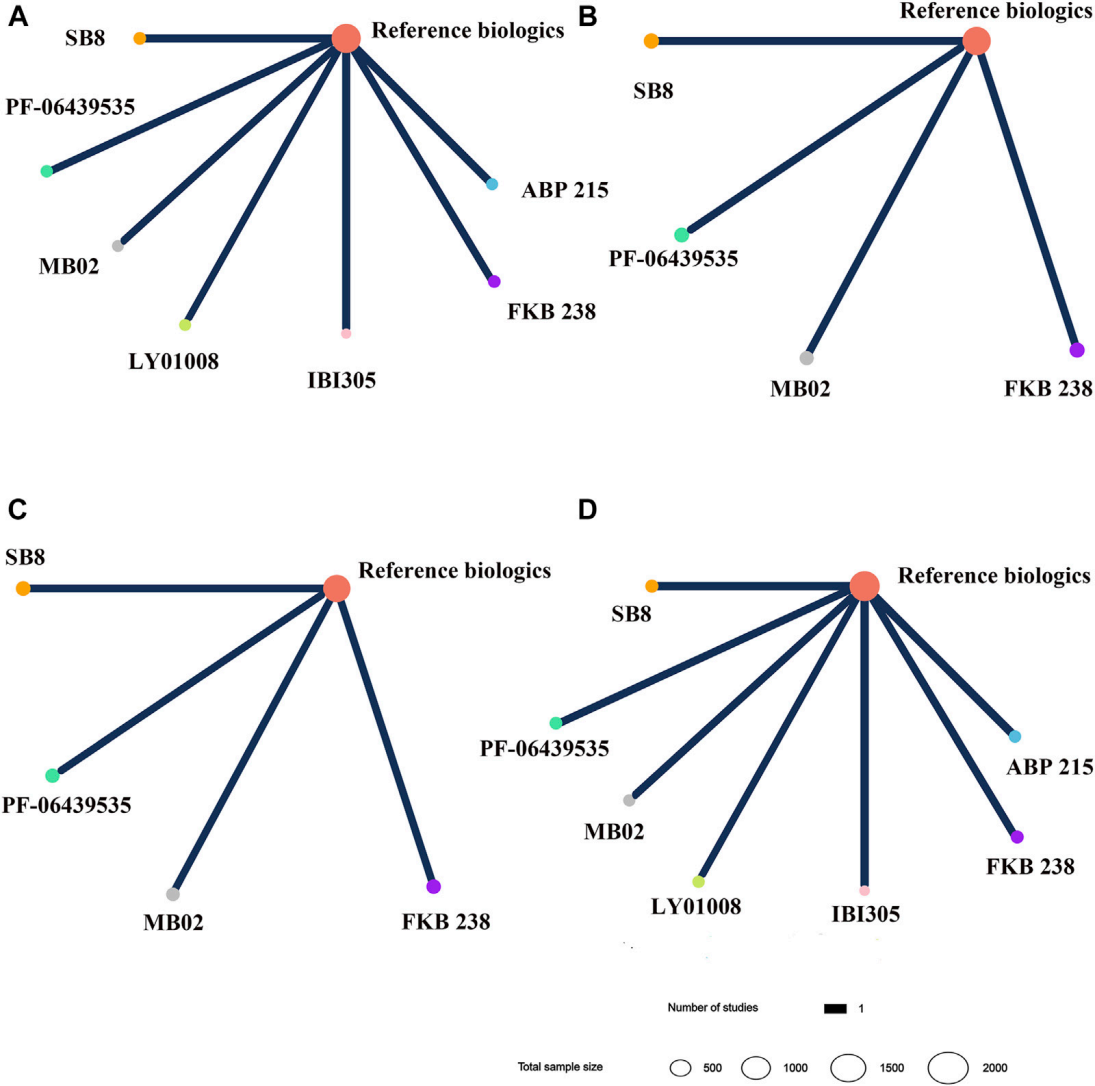
Network plots for different biosimilars and reference biologics in non-small cell lung cancer patients. **(A)** Objective response rate. **(B)** Progression-free survival. **(C)** Overall survival. **(D)** Incidence of grade 3–5 adverse events. Different colors of nodes indicated different treatments. The size of nodes corresponded to the number of participants investigating treatment. The thickness of the edge represented the number of trials. The lack of lines suggested that there are no head-to-head trials for this outcome between the two treatments.

**FIGURE 6:**
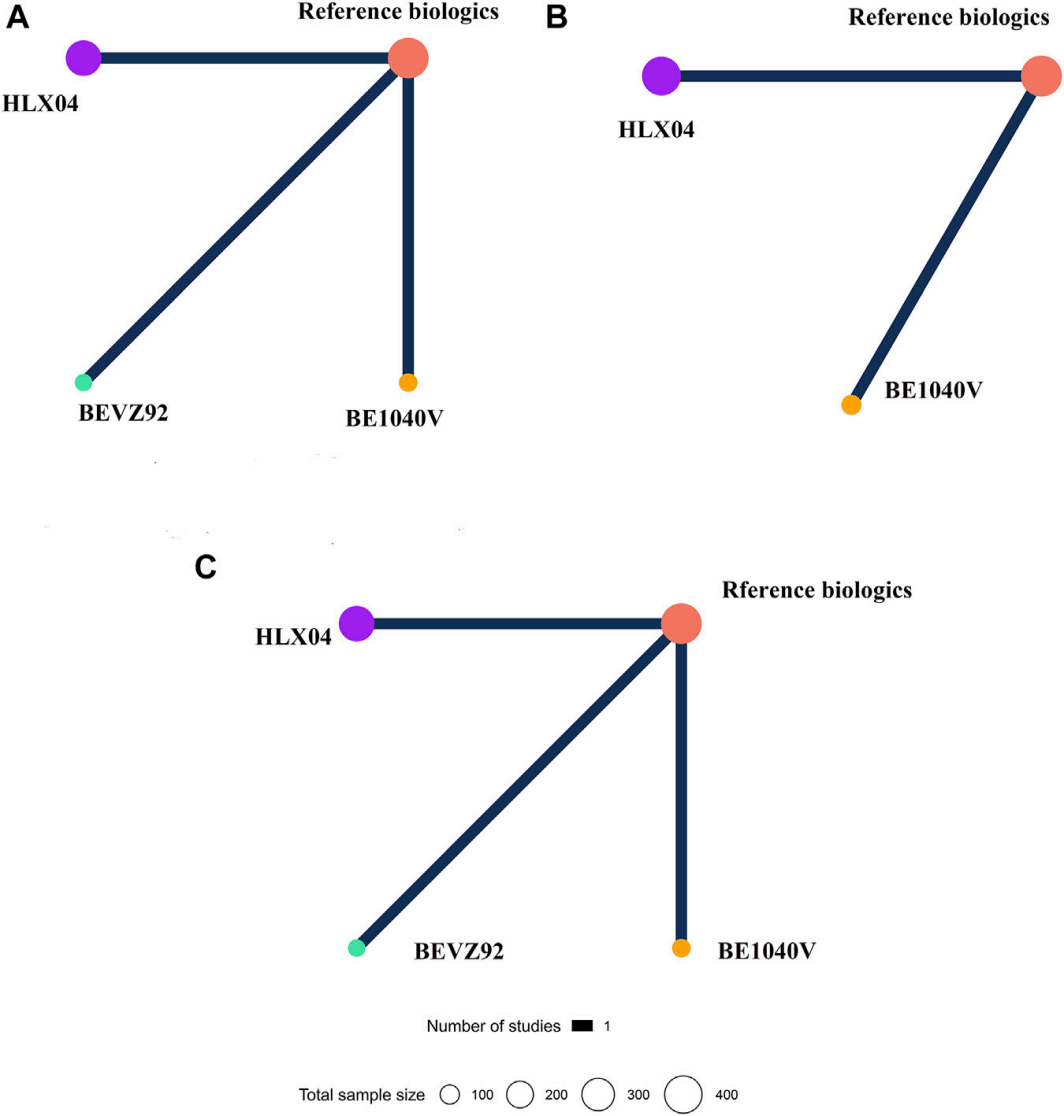
Network plots for different biosimilars and reference biologics in colorectal cancer patients. **(A)** Objective response rate. **(B)** Overall survival. **(C)** Incidence rate of grade 3–5 adverse events. Different colors of nodes indicated different treatments. The size of nodes corresponded to the number of participants investigating treatment. The thickness of the edge represented the number of trials. The lack of lines suggested that there are no head-to-head trials for this outcome between the two treatments.

**FIGURE 7:**
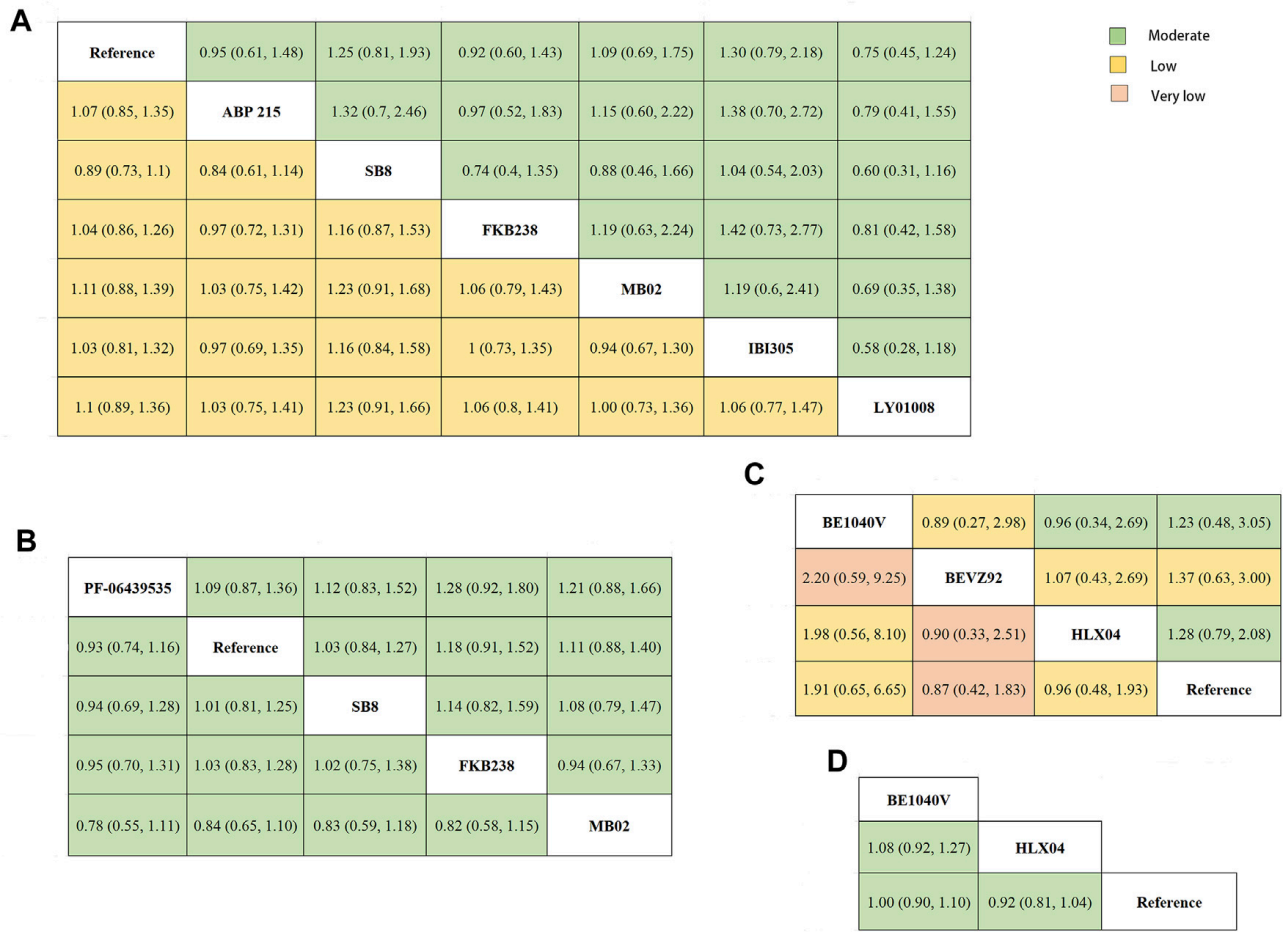
League table. **(A)** The network estimate of objective response rate (lower-left) and the incidence rate of grade 3–5 adverse events (upper-right) in patients with non-small cell lung cancer. **(B)** The network estimate of progression-free survival (lower-left) and overall survival (upper-right) in patients with non-small cell lung cancer. **(C)** The network estimate of objective response rate (lower-left) and the incidence rate of grade 3–5 adverse events (upper-right) in colorectal cancer patients. **(D)** The network estimate of overall survival in colorectal cancer patients. The league table presented the relative effects of each biosimilar and reference biologics (the treatment on the column to the treatment of the row). The relative effects were measured as risk ratio for objective response rate, odds ratios for incidence of grade 3–5 adverse events, and hazard ratio for progression-free survival and overall survival. The color of each cell indicated the certainty of evidence according to the Grading of Recommendations Assessment, Development, and Evaluation: green for moderate certainty, yellow for low certainty, and orange for very low certainty.

The network plots for all outcomes of different targeted patients are identified in Figure 5 and Figure 6. Figure 7 shows the results of the NMA and certainty of evidence for all estimates. We have summarized the detailed results of the certainty of the evidence for all comparisons in Supplementary Tables S2 and S3. According to the network plots, there was no node split analysis of our NMA due to no loop.

Concerning NSCLC, a total of seven studies involved in eight treatments were assessed for ORR (Figure 5A) (Reinmuth et al., 2019; Thatcher et al., 2019; Yunpeng Yang et al., 2019; Reck et al., 2020; Shi et al., 2021; Syrigos et al., 2021; Trukhin et al., 2021). The results of NMA presented that no significant difference was observed in ORR among any bevacizumab biosimilar for NSCLC patients, which was evaluated with low certainty (Figure 7A). Four studies reported the data on PFS and OS (Figure 5B, C) (Reinmuth et al., 2019; Reck et al., 2020; Syrigos et al., 2021; Trukhin et al., 2021). SB8, PF-06439535, MB02, and FKB238 were found to exist clinically equivalent survival benefits (moderate certainty; Figure 7B). As for safe outcomes, we saw no difference regarding the incidence of grade 3–5 AEs, which involved eight medication treatments (Figure 5D), in any comparable biosimilar therapy for the patients with NSCLC (moderate certainty; Figure 7A) (Reinmuth et al., 2019; Thatcher et al., 2019; Yunpeng Yang et al., 2019; Reck et al., 2020; Shi et al., 2021; Syrigos et al., 2021; Trukhin et al., 2021). Therefore, based on the existing evidence, seven biosimilars, including PF-06439535, ABP215, SB8, FKB238, MB02, LY01008, and IBI305, may have equivalent clinical efficacy and safety to each other in patients with NSCLC by multiple comparisons.

In terms of CRC, four treatments (BE1040V, BEVZ92, HLX04, and reference biologics) were evaluated for ORR (Figure 6A) (Romera et al., 2018; Rezvani et al., 2020; Qin et al., 2021). Based on the current published data, there was no significant difference in ORR among any treatment (from very low to low certainty; Figure 7C). In corresponding to OS, two studies published relevant data (Figure 6B) (Rezvani et al., 2020; Qin et al., 2021). According to the results of NMA, patients receiving the treatment of BE1040V, HLX04, and reference biologic had no significantly different survival benefits from each other (moderate certainty; Figure 7D). Also, the incidence of grade 3 or higher AEs (Figure 6C) was found to be comparable in BE1040V, BEVZ92, and HLX04 (from low to moderate certainty; Figure 7C) (Romera et al., 2018; Rezvani et al., 2020; Qin et al., 2021). Hence, BE1040V, BEVZ92, and HLX04 may be equivalent to each other in the population of CRC.

#### 3.4.2 Rank-Heat Plot Based on SUCRA

**FIGURE 8:**
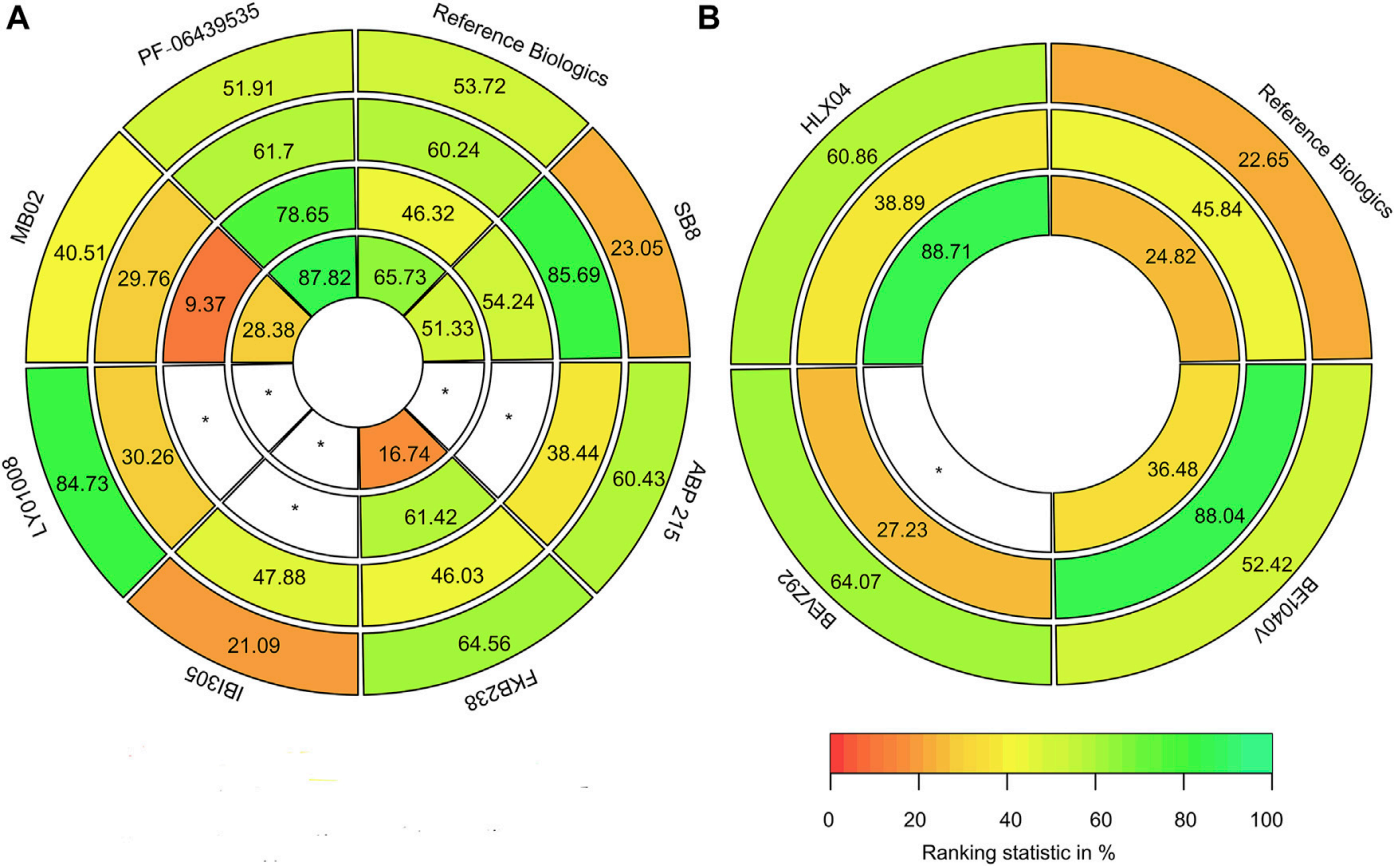
Rank-heat plot based on the surface under the cumulative ranking curve (SUCRA). **(A)** For non-small cell lung cancer patients, the index with the outer ring to the inner ring was the incidence rate of grade 3–5 adverse events, objective response rate, progression-free survival, and overall survival. **(B)** For colorectal cancer patients, the index with the outer ring to the inner ring was the incidence rate of grade 3–5 adverse events, objective response rate, progression-free survival, and overall survival. The scale comprises three color transformations: red (0%), yellow (50%), and green (100%), each color corresponding to a different evaluation indicator. The digits of sectors represented the SUCRA of all the outcomes. White sectors including a * represented treatments without data on the outcomes within the circle.

The rank-heat plot based on SUCRA is presented in Figure 8. Based on available published data, although this plot displayed that there might be subtle differences in SUCRA between the safety and efficacy of these biosimilars and reference biologics, there was no significant statistical difference among them.

## 4 Discussion

Our study provides an overview of evidence regarding the efficacy and safety of different bevacizumab biosimilars in the treatment of patients with NSCLC or CRC. According to the findings of the pairwise meta-analysis, the efficacy and safety of bevacizumab biosimilars in existence currently were not substantially different from the reference biologics. Furthermore, the results of NMA demonstrated that there was no significant difference between different bevacizumab biosimilars and each other. We also assessed immunogenicity (Supplementary Appendix S4) and found no significant difference. The results of our study can provide a reference for clinicians and payers when facing different biosimilars in the treatment of patients with NSCLC or CRC.

In our study, ten RCTs that we included were all taking the bevacizumab reference biologics (Avastin^®^) as the control group, and the number of the study regarding every biosimilar was only one; thus, heterogeneity among studies was unable to analyze. Nevertheless, we had got heterogeneity in the pairwise meta-analysis by regarding all the biosimilars as a category in two specific diseases. According to forest plots of pairwise meta-analysis, the heterogeneities of all outcomes were completely not more than 20%; therefore, we considered the heterogeneity in our NMA to be acceptable. Considering that the heterogeneity was acceptable, we also did not need to undertake further sensitivity analysis. Moreover, the transitivity of our results was acceptable, as we screened strictly according to our inclusion and exclusion criteria for leading to a comparable baseline of participation. Node splitting analysis could not be conducted because of no loop in our NMA; thus, consistency assessment could not be accomplished.

In recent years, a variety of clinical trials and meta-analysis regarding biosimilars are focusing more on chronic illnesses (Nast et al., 2015; Hanrahan and Lee, 2021; Safdar et al., 2021). Nevertheless, with the widespread emergence and application of biosimilars in the field of tumors, relevant studies have gradually emerged (Botteri et al., 2018; Cargnin et al., 2020). Several scholars mainly used classic meta-analysis to focus on comparisons between biosimilars and reference biologics in cancer patients; thus, comparative evidence is still scarce among different biosimilars. (Botteri et al., 2018; Cargnin et al., 2020). To a certain extent, our research can fill this gap.

Before our study, only one meta-analysis focused on bevacizumab biosimilars, thereby revealing that bevacizumab biosimilars had no clinically significant differences in safety, efficacy, and immunogenicity with reference biologics, which was also consistent with our findings (Jichun Yang et al., 2019). However, the subgroup analysis of specific cancer types in that review was hampered by lack of data and insufficient evidence (Jichun Yang et al., 2019). To the best of our knowledge, our NMA is the first Bayesian NMA to focus on different bevacizumab biosimilars in patients with NSCLC or CRC. Our study is not only an update of the data of the meta-analysis above but also summarizes the evidence for comparisons between different bevacizumab biosimilars. At present, the evaluation of bevacizumab biosimilars in real-world settings is still limited. Kumar et al. found that both bevacizumab reference biologics and biosimilar seemed to have similar safety and clinical efficacy in the recurrent or progressive glioblastoma patients (Kumar et al., 2021). Until now, since there are currently no real-world studies on NSCLC or CRC, it may be necessary to encourage more researchers to establish large real-world studies with long-term follow-up to further verify the safety and efficacy of bevacizumab biosimilars in the real-world environment in the future. Our study also provides some preliminary evidence to support future observational studies.

There are still some limitations to this review. First, we only included premarketing clinical studies (phase III RCTs). Clinical trials have strict inclusion and exclusion criteria and may not truly reflect real-world conditions due to the complexity of clinical patients and settings. Therefore, the results of our research may not be extended to the real-world environment, and more studies based on real-world data may be needed to further validate the findings of this review. Second, the data on CRC are still relatively poor, and the treatment regimens used in each trial are not uniform. Thus, we had no way of subgroup analysis using chemotherapy regimen. Third, interchangeability is an important issue that must be considered for biosimilars. However, no relevant studies were found during our search. Therefore, this issue has not been discussed in external studies.

## 5 Conclusion

The clinical efficacy and safety of different bevacizumab biosimilars were comparable with each other in advanced NSCLC or CRC patients. Overall, our findings further support the utilization of biosimilars in clinical practice. More future studies, especially real-world studies, are needed to further corroborate the results of our analysis.

## Data Availability

The original contributions presented in the study are included in the article/Supplementary Material; further inquiries can be directed to the corresponding author.
