# Supplementary material for: Efficacy and Safety of Bevacizumab Biosimilars Compared With Reference Biologics in Advanced Non-small Cell Lung Cancer or Metastatic Colorectal Cancer Patients: A Network Meta-Analysis
Source: Front Pharmacol. 2022 Jul 5;13:880090. doi: 10.3389/fphar.2022.880090 (PMC9294356; doi:10.3389/fphar.2022.880090)
Supplement: Supplementary file 1 [file DataSheet1.docx]

Supplementary Material

**Content:**

**Supplementary Materials**

**Supplementary Appendix 1:** PRISMA 2020 checklist for meta-analysis

**Supplementary Appendix 2:** PRISMA checklist for network meta-analysis

**Supplementary Appendix 3:** Search strategy

**Supplementary Appendix 4:** Immunogencity

4.1 The results of pari-wise meta-analysis for incidence of anti-drug antibodies

4.1.1 Forest plot for patients with non-small cell lung cancer

4.1.2 Forest plot for patients with colorectal cancer

4.1.3 Funnel plot for incidence of anti-drug antibodies.

**(A)** Non-small cell lung cancer; **(B)** Colorectal cancer

4.2 The results of network meta-analysis for incidence of anti-drug antibodies

4.2.1 Network plot for non-small cell lung cancer

4.2.2 Network plot for colorectal cancer

4.2.3 Network estimates

4.2.4 Surface under the cumulative ranking curve (SUCRA)

**Supplementary Table S1:** Assessment of the quality of evidence for pair-wise meta-analysis

**Supplementary Table S2:** Certainty of evidence for direct, indirect and network estimates about non-small cell lung cancer patients

**Supplementary Table S3:** Certainty of evidence for direct, indirect and network estimates about colorectal cancer patients

**Supplementary Figure S1:** Risk of bias assessment

**Supplementary Figure S2:** Funnel plot for non-small cell lung cancer patients

**(A)** Objective response rate; **(B)** Progression-free survival; **(C)** Overall survival; **(D)** Incidence of grade 3-5 adverse events

**Supplementary Figure S3:** Funnel plot for colorectal cancer patients

**(A)** Objective response rate; **(B)** Overall survival; **(C)** Incidence of grade 3-5 adverse events

**Supplementary Appendix 1:** PRISMA 2020 checklist for meta-analysis

| **Section and Topic** | **Item #** | **Checklist item** | **Location where item is reported** |
| --- | --- | --- | --- |
| **TITLE** | | |  |
| Title | 1 | Identify the report as a systematic review. | Page 1 |
| **ABSTRACT** | | |  |
| Abstract | 2 | See the PRISMA 2020 for Abstracts checklist. | Page 1-2 |
| **INTRODUCTION** | | |  |
| Rationale | 3 | Describe the rationale for the review in the context of existing knowledge. | Page 2 |
| Objectives | 4 | Provide an explicit statement of the objective(s) or question(s) the review addresses. | Page 2 |
| **METHODS** | | |  |
| Eligibility criteria | 5 | Specify the inclusion and exclusion criteria for the review and how studies were grouped for the syntheses. | Page 2-3 |
| Information sources | 6 | Specify all databases, registers, websites, organisations, reference lists and other sources searched or consulted to identify studies. Specify the date when each source was last searched or consulted. | Page 2 |
| Search strategy | 7 | Present the full search strategies for all databases, registers and websites, including any filters and limits used. | Supplementary Appendix 3 |
| Selection process | 8 | Specify the methods used to decide whether a study met the inclusion criteria of the review, including how many reviewers screened each record and each report retrieved, whether they worked independently, and if applicable, details of automation tools used in the process. | Page 2-3 |
| Data collection process | 9 | Specify the methods used to collect data from reports, including how many reviewers collected data from each report, whether they worked independently, any processes for obtaining or confirming data from study investigators, and if applicable, details of automation tools used in the process. | Page 3 |
| Data items | 10a | List and define all outcomes for which data were sought. Specify whether all results that were compatible with each outcome domain in each study were sought (e.g. for all measures, time points, analyses), and if not, the methods used to decide which results to collect. | Page 2-3 |
|  | 10b | List and define all other variables for which data were sought (e.g. participant and intervention characteristics, funding sources). Describe any assumptions made about any missing or unclear information. | Table 1 |
| Study risk of bias assessment | 11 | Specify the methods used to assess risk of bias in the included studies, including details of the tool(s) used, how many reviewers assessed each study and whether they worked independently, and if applicable, details of automation tools used in the process. | Page 3 |
| Effect measures | 12 | Specify for each outcome the effect measure(s) (e.g. risk ratio, mean difference) used in the synthesis or presentation of results. | Page 3, 5 |
| Synthesis methods | 13a | Describe the processes used to decide which studies were eligible for each synthesis (e.g. tabulating the study intervention characteristics and comparing against the planned groups for each synthesis (item #5)). | Page 2-3, 5 |
|  | 13b | Describe any methods required to prepare the data for presentation or synthesis, such as handling of missing summary statistics, or data conversions. | Page 3, 5 |
|  | 13c | Describe any methods used to tabulate or visually display results of individual studies and syntheses. | Page 3, 5 |
|  | 13d | Describe any methods used to synthesize results and provide a rationale for the choice(s). If meta-analysis was performed, describe the model(s), method(s) to identify the presence and extent of statistical heterogeneity, and software package(s) used. | Page 3, 5 |
|  | 13e | Describe any methods used to explore possible causes of heterogeneity among study results (e.g. subgroup analysis, meta-regression). | Page 3, 5 |
|  | 13f | Describe any sensitivity analyses conducted to assess robustness of the synthesized results. | Not applicable |
| Reporting bias assessment | 14 | Describe any methods used to assess risk of bias due to missing results in a synthesis (arising from reporting biases). | Page 3 |
| Certainty assessment | 15 | Describe any methods used to assess certainty (or confidence) in the body of evidence for an outcome. | Page 5 |
| **RESULTS** | | |  |
| Study selection | 16a | Describe the results of the search and selection process, from the number of records identified in the search to the number of studies included in the review, ideally using a flow diagram. | Page 5  Supplementary Appendix 3  Figure 1 |
|  | 16b | Cite studies that might appear to meet the inclusion criteria, but which were excluded, and explain why they were excluded. | Page 5-6  Figure 1 |
| Study characteristics | 17 | Cite each included study and present its characteristics. | Page 6  Table 1 |
| Risk of bias in studies | 18 | Present assessments of risk of bias for each included study. | Page 6-7  Supplementary Figure S1 |
| Results of individual studies | 19 | For all outcomes, present, for each study: (a) summary statistics for each group (where appropriate) and (b) an effect estimate and its precision (e.g. confidence/credible interval), ideally using structured tables or plots. | Page 7-8 |
| Results of syntheses | 20a | For each synthesis, briefly summarise the characteristics and risk of bias among contributing studies. | Page 6-8 |
|  | 20b | Present results of all statistical syntheses conducted. If meta-analysis was done, present for each the summary estimate and its precision (e.g. confidence/credible interval) and measures of statistical heterogeneity. If comparing groups, describe the direction of the effect. | Page 7-8 |
|  | 20c | Present results of all investigations of possible causes of heterogeneity among study results. | Page 7-8 |
|  | 20d | Present results of all sensitivity analyses conducted to assess the robustness of the synthesized results. | Not applicable |
| Reporting biases | 21 | Present assessments of risk of bias due to missing results (arising from reporting biases) for each synthesis assessed. | Supplementary Figure S2, S3 |
| Certainty of evidence | 22 | Present assessments of certainty (or confidence) in the body of evidence for each outcome assessed. | Page 7-8  Supplementary Table S1 |
| **DISCUSSION** | | |  |
| Discussion | 23a | Provide a general interpretation of the results in the context of other evidence. | Page 10 |
|  | 23b | Discuss any limitations of the evidence included in the review. | Page 11 |
|  | 23c | Discuss any limitations of the review processes used. | Page 11 |
|  | 23d | Discuss implications of the results for practice, policy, and future research. | Page 10-11 |
| **OTHER INFORMATION** | | |  |
| Registration and protocol | 24a | Provide registration information for the review, including register name and registration number, or state that the review was not registered. | Page 2 |
|  | 24b | Indicate where the review protocol can be accessed, or state that a protocol was not prepared. | Page 2 |
|  | 24c | Describe and explain any amendments to information provided at registration or in the protocol. | Page 2 |
| Support | 25 | Describe sources of financial or non-financial support for the review, and the role of the funders or sponsors in the review. | Page 12 |
| Competing interests | 26 | Declare any competing interests of review authors. | Page 13 |
| Availability of data, code and other materials | 27 | Report which of the following are publicly available and where they can be found: template data collection forms; data extracted from included studies; data used for all analyses; analytic code; any other materials used in the review. | Not applicable |

**Supplementary Appendix 2:** PRISMA checklist for NMA

| **Section/Topic** | **Item #** | **Checklist Item** | **Reported on Page #** |
| --- | --- | --- | --- |
| **TITLE** |  |  |  |
| Title | 1 | Identify the report as a systematic review *incorporating a network meta-analysis (or related form of meta-analysis).* | Page 1 |
|  |  |  |  |
| **ABSTRACT** |  |  |  |
| Structured summary | 2 | Provide a structured summary including, as applicable:  **Background:** main objectives  **Methods:** data sources; study eligibility criteria, participants, and interventions; study appraisal; and *synthesis methods, such as network meta-analysis.*  **Results:** number of studies and participants identified; summary estimates with corresponding confidence/credible intervals; *treatment rankings may also be discussed. Authors may choose to summarize pairwise comparisons against a chosen treatment included in their analyses for brevity.*  **Discussion/Conclusions:** limitations; conclusions and implications of findings.  **Other:** primary source of funding; systematic review registration number with registry name. | Page 1-2 |
|  |  |  |  |
| **INTRODUCTION** |  |  |  |
| Rationale | 3 | Describe the rationale for the review in the context of what is already known*, including mention of why a network meta-analysis has been conducted.* | Page 2 |
| Objectives | 4 | Provide an explicit statement of questions being addressed, with reference to participants, interventions, comparisons, outcomes, and study design (PICOS). | Page 2 |
|  |  |  |  |
| **METHODS** |  |  |  |
| Protocol and registration | 5 | Indicate whether a review protocol exists and if and where it can be accessed (e.g., Web address); and, if available, provide registration information, including registration number. | Page 2 |
| Eligibility criteria | 6 | Specify study characteristics (e.g., PICOS, length of follow-up) and report characteristics (e.g., years considered, language, publication status) used as criteria for eligibility, giving rationale. *Clearly describe eligible treatments included in the treatment network, and note whether any have been clustered or merged into the same node (with justification).* | Page 2-3 |
| Information sources | 7 | Describe all information sources (e.g., databases with dates of coverage, contact with study authors to identify additional studies) in the search and date last searched. | Page 2 |
| Search | 8 | Present full electronic search strategy for at least one database, including any limits used, such that it could be repeated. | Supplementary Appendix 3 |
| Study selection | 9 | State the process for selecting studies (i.e., screening, eligibility, included in systematic review, and, if applicable, included in the meta-analysis). | Page 3 |
| Data collection process | 10 | Describe method of data extraction from reports (e.g., piloted forms, independently, in duplicate) and any processes for obtaining and confirming data from investigators. | Page 3 |
| Data items | 11 | List and define all variables for which data were sought (e.g., PICOS, funding sources) and any assumptions and simplifications made. | Page 2-3  Table 1 |
| **Geometry of the network** | **S1** | Describe methods used to explore the geometry of the treatment network under study and potential biases related to it. This should include how the evidence base has been graphically summarized for presentation, and what characteristics were compiled and used to describe the evidence base to readers. | Page 3 |
| Risk of bias within individual studies | 12 | Describe methods used for assessing risk of bias of individual studies (including specification of whether this was done at the study or outcome level), and how this information is to be used in any data synthesis. | Page 3 |
| Summary measures | 13 | State the principal summary measures (e.g., risk ratio, difference in means). *Also describe the use of additional summary measures assessed, such as treatment rankings and surface under the cumulative ranking curve (SUCRA) values, as well as modified approaches used to present summary findings from meta-analyses.* | Page 3, 5 |
| Planned methods of analysis | 14 | Describe the methods of handling data and combining results of studies for each network meta-analysis. This should include, but not be limited to:   - *Handling of multi-arm trials;* - *Selection of variance structure;* - *Selection of prior distributions in Bayesian analyses; and* - *Assessment of model fit.* | Page 3, 5 |
| **Assessment of Inconsistency** | **S2** | Describe the statistical methods used to evaluate the agreement of direct and indirect evidence in the treatment network(s) studied. Describe efforts taken to address its presence when found. | Page 5 |
| Risk of bias across studies | 15 | Specify any assessment of risk of bias that may affect the cumulative evidence (e.g., publication bias, selective reporting within studies). | Page 3 |
| Additional analyses | 16 | Describe methods of additional analyses if done, indicating which were pre-specified. This may include, but not be limited to, the following:   - Sensitivity or subgroup analyses; - Meta-regression analyses; - *Alternative formulations of the treatment network; and* - *Use of alternative prior distributions for Bayesian analyses (if applicable).* | Not applicable |
|  |  |  |  |
| **RESULTS†** |  |  |  |
| Study selection | 17 | Give numbers of studies screened, assessed for eligibility, and included in the review, with reasons for exclusions at each stage, ideally with a flow diagram. | Page 5-6  Supplementary Appendix 3  Figure 1 |
| **Presentation of network structure** | **S3** | Provide a network graph of the included studies to enable visualization of the geometry of the treatment network. | Figure 5 and 6 |
| **Summary of network geometry** | **S4** | Provide a brief overview of characteristics of the treatment network. This may include commentary on the abundance of trials and randomized patients for the different interventions and pairwise comparisons in the network, gaps of evidence in the treatment network, and potential biases reflected by the network structure. | Page 8-9 |
| Study characteristics | 18 | For each study, present characteristics for which data were extracted (e.g., study size, PICOS, follow-up period) and provide the citations. | Page 5-6  Table 1 |
| Risk of bias within studies | 19 | Present data on risk of bias of each study and, if available, any outcome level assessment. | Page 5  Supplementary Figure S1 |
| Results of individual studies | 20 | For all outcomes considered (benefits or harms), present, for each study: 1) simple summary data for each intervention group, and 2) effect estimates and confidence intervals. *Modified approaches may be needed to deal with information from larger networks.* | Page 8-9 |
| Synthesis of results | 21 | Present results of each meta-analysis done, including confidence/credible intervals. *In larger networks, authors may focus on comparisons versus a particular comparator (e.g. placebo or standard care), with full findings presented in an appendix. League tables and forest plots may be considered to summarize pairwise comparisons.* If additional summary measures were explored (such as treatment rankings), these should also be presented. | Page 8-9 |
| **Exploration for inconsistency** | **S5** | Describe results from investigations of inconsistency. This may include such information as measures of model fit to compare consistency and inconsistency models, *P* values from statistical tests, or summary of inconsistency estimates from different parts of the treatment network. | Page 8-9 |
| Risk of bias across studies | 22 | Present results of any assessment of risk of bias across studies for the evidence base being studied. | Supplementary Figure S2, S3 |
| Results of additional analyses | 23 | Give results of additional analyses, if done (e.g., sensitivity or subgroup analyses, meta-regression analyses*, alternative network geometries studied, alternative choice of prior distributions for Bayesian analyses,* and so forth). | Not applicable |
|  |  |  |  |
| **DISCUSSION** |  |  |  |
| Summary of evidence | 24 | Summarize the main findings, including the strength of evidence for each main outcome; consider their relevance to key groups (e.g., healthcare providers, users, and policy-makers). | Page 10 |
| Limitations | 25 | Discuss limitations at study and outcome level (e.g., risk of bias), and at review level (e.g., incomplete retrieval of identified research, reporting bias). *Comment on the validity of the assumptions, such as transitivity and consistency. Comment on any concerns regarding network geometry (e.g., avoidance of certain comparisons).* | Page 11 |
| Conclusions | 26 | Provide a general interpretation of the results in the context of other evidence, and implications for future research. | Page 11-12 |
|  |  |  |  |
| **FUNDING** |  |  |  |
| Funding | 27 | Describe sources of funding for the systematic review and other support (e.g., supply of data); role of funders for the systematic review. This should also include information regarding whether funding has been received from manufacturers of treatments in the network and/or whether some of the authors are content experts with professional conflicts of interest that could affect use of treatments in the network. | Page 12 |

**Supplementary Appendix 3:** Search strategy

| **Search strategy** | | **Number of studies** |
| --- | --- | --- |
| **Pubmed** | | |
| #1 | “Biosimilar Pharmaceuticals”[Mesh] OR “Subsequent Entry Biologics”[Title/Abstract] OR “Follow-on Biologics” [Title/Abstract] OR “Biologics, Follow-on” [Title/Abstract] OR “Follow on Biologics” [Title/Abstract] OR “Biosimilars” OR “Biosimilar” [Title/Abstract] | 4,422 |
| #2 | "Bevacizumab"[Mesh] OR “Mvasi” [Title/Abstract] OR “Bevacizumab-awwb” [Title/Abstract] OR “Bevacizumab awwb” [Title/Abstract] OR “Avastin” [Title/Abstract] | 13,046 |
| #3 | #1 AND #2 | 83 |
| #4 | #3 Filters: Randomized Controlled Trial | 24 |
| **Embase (via Ovid)** | | |
| #1 | ‘biosimilar agent’/exp | 5452 |
| #2 | ‘bevacizumab’/ exp | 63,306 |
| #3 | #1 AND #2 AND 'randomized controlled trial'/exp | 291 |
| **Cochrane (via Ovid)** | | |
| #1 | ‘Biosimilar Pharmaceuticals’.mp | 235 |
| #2 | ‘Bevacizumab’.mp | 7,404 |
| #3 | #1 and #2 | 24 |
| **Medline (via Ovid)** | | |
| #1 | ‘Bevacizumab’ | 12,547 |
| #2 | ‘Biosimilar Pharmaceuticals’ | 2,530 |
| #3 | #1and #2 | 62 |

**Supplementary Appendix 4:** Immunogencity

4.1 The results of pari-wise meta-analysis for incidence of anti-drug antibodies

4.1.1 Forest plot for patients with non-small cell lung cancer


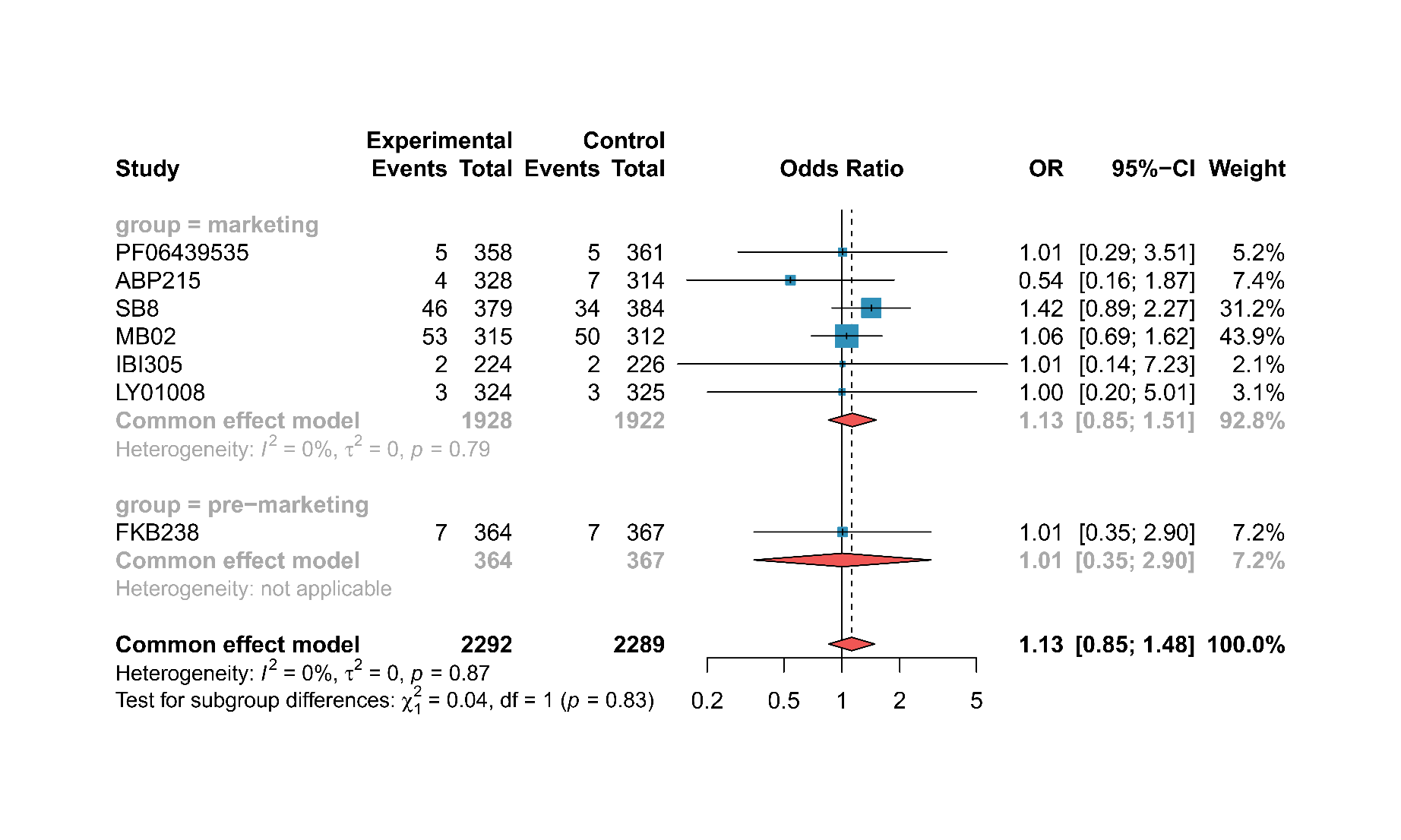


The incidence of anti-drug antibodies was measured by odds ratio (OR). If the OR value is higher than 1, it favoured the reference biologics group; on the contrary, it favoured the biosimilars group. CI, confidence interval; OR, odds ratio

4.1.2 Forest plot for patients with colorectal cancer


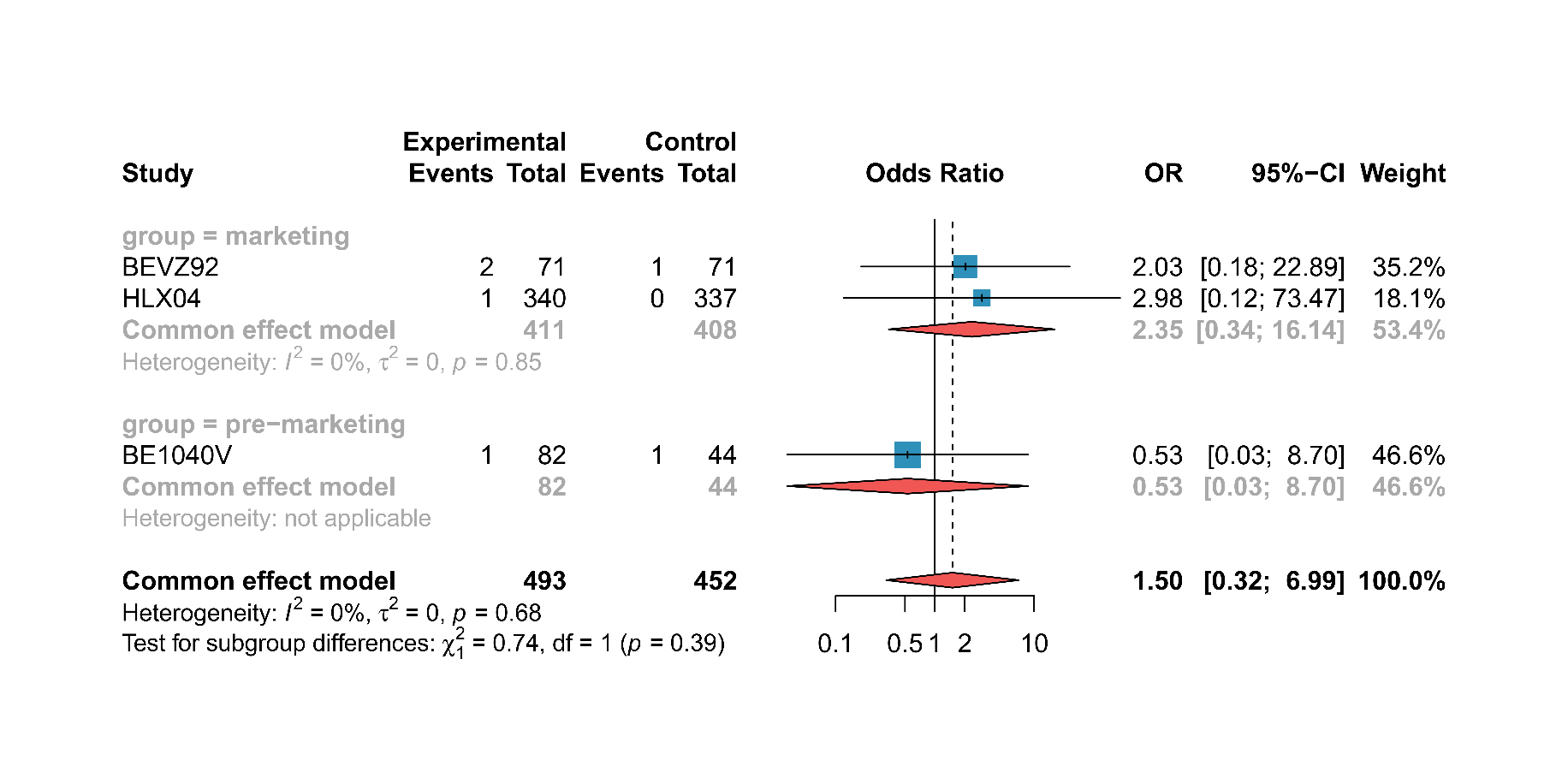


The incidence of anti-drug antibodies was measured by odds ratio (OR). If the OR value is higher than 1, it favoured the reference biologics group; on the contrary, it favoured the biosimilars group. CI, confidence interval; OR, odds ratio

4.1.3 Funnel plot for incidence of anti-drug antibodies.

**(A)** non-small cell lung cancer; **(B)** colorectal cancer


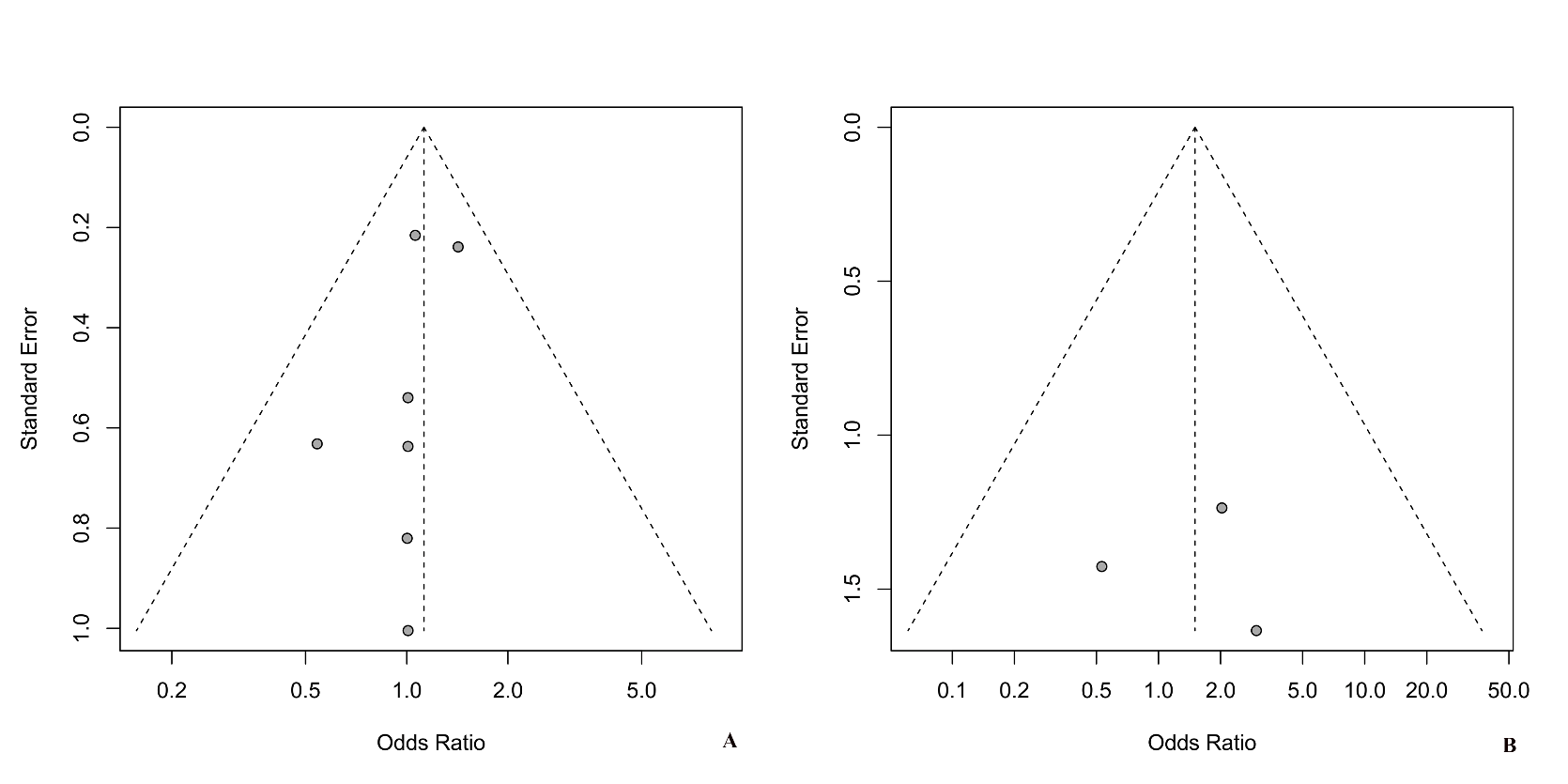


4.2 The results of network meta-analysis for incidence of anti-drug antibodies

4.2.1 Network plot for non-small cell lung cancer

**
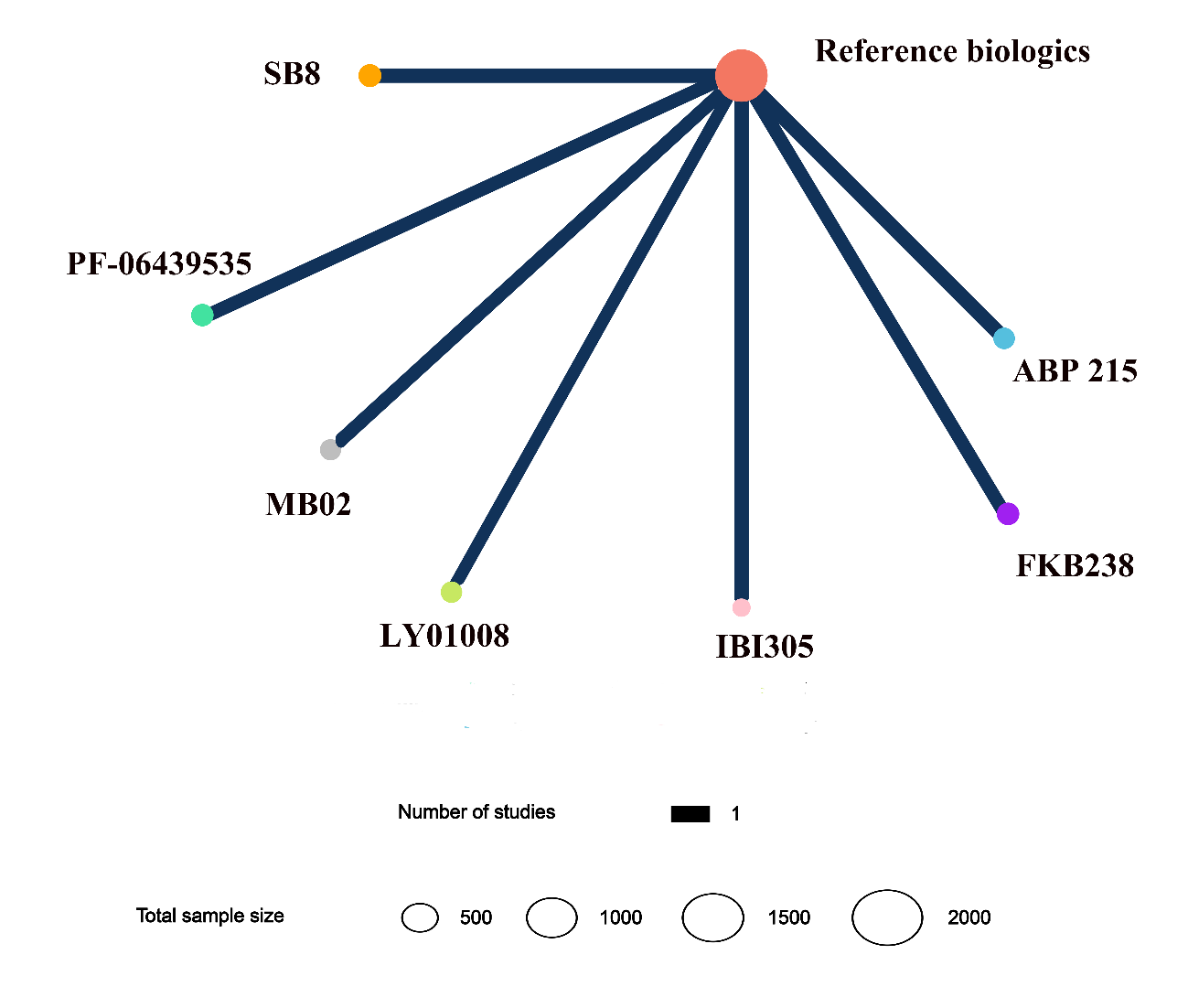
**

Different color of nodes indicated different treatments. The size of nodes corresponded to the number of participants investigating treatment. The thickness of the edge represented the number of trials. The lack of lines suggested that there are no head-to-head trials for this outcome between the two treatments.

4.2.2 Network plot for colorectal cancer


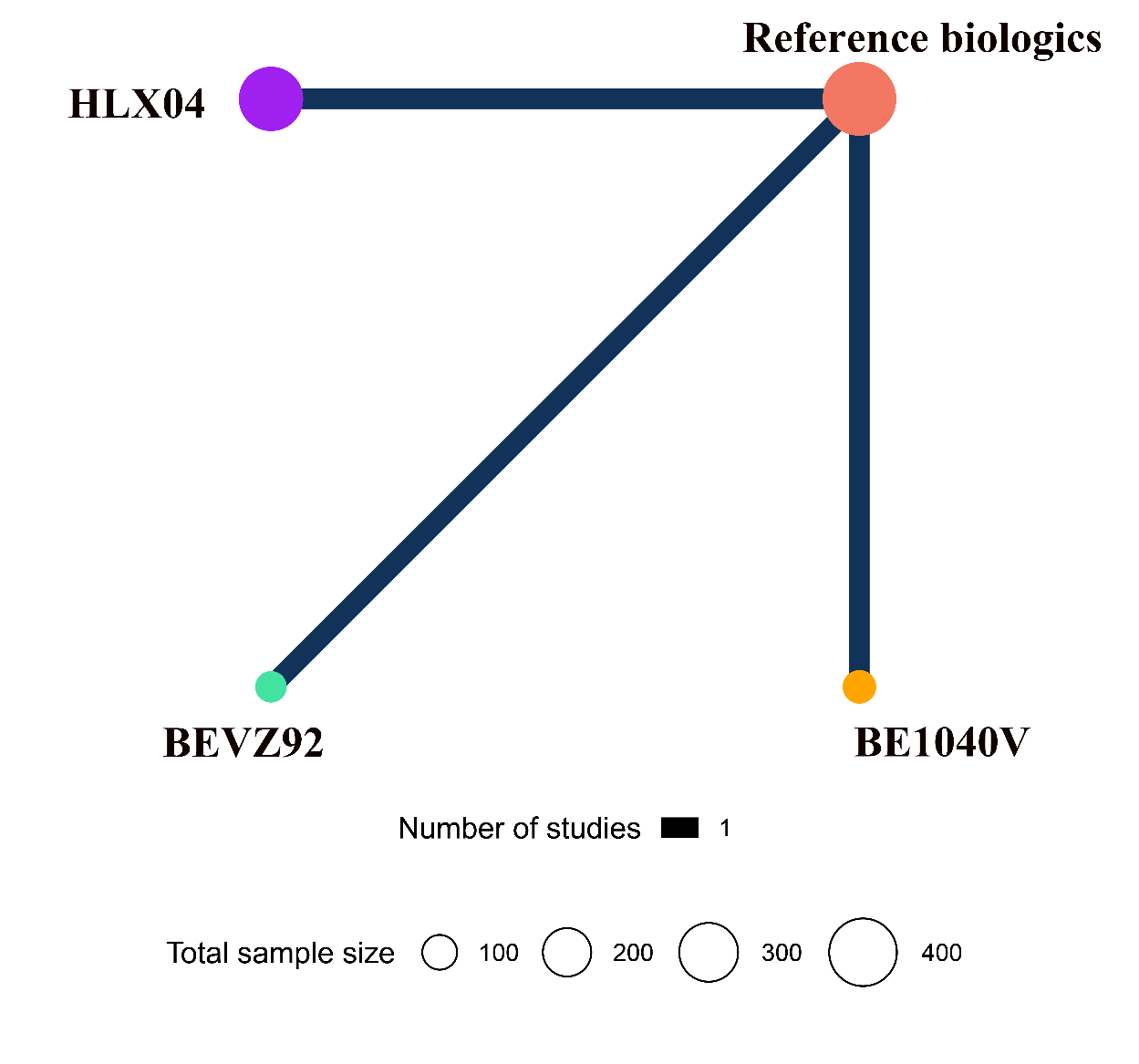


Different color of nodes indicated different treatments. The size of nodes corresponded to the number of participants investigating treatment. The thickness of the edge represented the number of trials. The lack of lines suggested that there are no head-to-head trials for this outcome between the two treatments.

4.2.3 Network estimates

| **Comparisons** | **network estmates** | **Comparisons** | | **network estmates** |
| --- | --- | --- | --- | --- |
| **Non-small cell lung cancer** |  | |  |  |
| ABP 215 vs. FKB238 | 0.53 (0.08, 3.51) | | FKB238 vs. IBI305 | 0.96 (0.07, 13.6) |
| ABP 215 vs. IBI305 | 0.51 (0.03, 7.78) | | FKB238 vs. LY01008 | 0.97 (0.1, 9.31) |
| ABP 215 vs. LY01008 | 0.51 (0.05, 5.36) | | FKB238 vs. MB02 | 0.96 (0.21, 4.25) |
| ABP 215 vs. MB02 | 0.51 (0.09, 2.51) | | FKB238 vs. PF-06439535 | 0.99 (0.14, 6.9) |
| ABP 215 vs. PF-06439535 | 0.53 (0.07, 3.99) | | FKB238 vs. Reference biologics | 1.01 (0.28, 3.6) |
| ABP 215 vs. Refernence biologics | 0.54 (0.12, 2.16) | | FKB238 vs. SB8 | 0.71 (0.16, 3.21) |
| ABP 215 vs. SB8 | 0.38 (0.07, 1.88) | | IBI305 vs. LY01008 | 1.01 (0.05, 20.29) |
| LY01008 vs. MB02 | 0.98 (0.13, 7.47) | | IBI305 vs. MB02 | 0.99 (0.09, 11.99) |
| LY01008 vs. PF-06439535 | 1.03 (0.1, 11.05) | | IBI305 vs. PF-06439535 | 1.03 (0.07, 16.32) |
| LY01008 vs. Reference biologics | 1.05 (0.16, 6.78) | | IBI305 vs. Reference biologics | 1.05 (0.1, 11.27) |
| LY01008 vs. SB8 | 0.73 (0.09, 5.54) | | IBI305 vs. SB8 | 0.74 (0.06, 9.06) |
| MB02 vs. PF-06439535 | 1.04 (0.2, 5.48) | | PF-06439535 vs. Reference biologics | 1.02 (0.23, 4.42) |
| MB02 vs. Reference biologics | 1.06 (0.47, 2.37) | | PF-06439535 vs. SB8 | 0.71 (0.13, 3.8) |
| MB02 vs. SB8 | 0.74 (0.23, 2.35) | | Reference biologics vs. SB8 | 0.7 (0.31, 1.59) |
|  |  | |  |  |
| **Coloractal cancer** |  | |  |  |
| BE1040V vs. BEVZ92 | 0.21 (0, 26.35) | | BEVZ92 vs. HLX04 | 0 (0, 8.4) |
| BE1040V vs. HLX04 | 0 (0, 2.13) | | BEVZ92 vs. Reference biologics | 2.47 (0.14, 89.07) |
|  |  | |  |  |
| BE1040V vs. Reference biologics | 0.55 (0.01, 27.08) | | HLX04 vs. Reference biologics | 52983.51 (0.66, 22511251451075592) |

4.2.4 SUCRA

| **Biosimilars** | **SUCRA** |
| --- | --- |
| **Non-small cell lung cancer** |  |
| ABP 215 | 0.7710 |
| FKB238 | 0.4960 |
| IBI305 | 0.4815 |
| LY01008 | 0.4818 |
| MB02 | 0.4714 |
| PF-06439535 | 0.4939 |
| Reference biologics | 0.5208 |
| SB8 | 0.2835 |
|  |  |
| **Coloractal cancer** |  |
| BE1040V | 0.7810 |
| BEVZ92 | 0.4822 |
| HLX04 | 0.0493 |
| Reference biologics | 0.6875 |

**Supplementary Table S1:** Assessment of the quality of evidence for pair-wise meta-analysis

| **Comparisions** | **Number of trials** | **Participants** | **Within-study risk of bias** | **Indirectness** | **Heterogeneity** | **Imprecision** | **Quality of evidence** |
| --- | --- | --- | --- | --- | --- | --- | --- |
| **Non-small cell lung cancer** |  |  |  |  |  |  |  |
| ORR |  |  |  |  |  |  |  |
| Biosimilars vs. Reference biologics | 7 | 4581 | No serious limitations: Other bias- unclear risk in 7 (100%) trials. | Indirect (-1) | No important heterogeneity;  I^2^=0% | Imprecise (-1) | Low |
| PFS |  |  |  |  |  |  |  |
| Biosimilars vs. Reference biologics | 7 | 4581 | No serious limitations: Other bias- unclear risk in 7 (100%) trials. | Direct | No important heterogeneity;  I^2^=20% | Imprecise (-1) | Moderate |
| OS |  |  |  |  |  |  |  |
| Biosimilars vs. Reference biologics | 7 | 4581 | No serious limitations: Other bias- unclear risk in 7 (100%) trials. | Direct | No important heterogeneity;  I^2^=0% | Imprecise (-1) | Moderate |
| Incidence of grade 3-5 AEs |  |  |  |  |  |  |  |
| Biosimilars vs. Reference biologics | 7 | 4581 | No serious limitations: Other bias- unclear risk in 7 (100%) trials. | Direct | No important heterogeneity;  I^2^=7% | Imprecise (-1) | Moderate |
|  |  |  |  |  |  |  |  |
| **Colorectal cancer** |  |  |  |  |  |  |  |
| ORR |  |  |  |  |  |  |  |
| Biosimilars vs. Reference biologics | 3. | 945 | Potential limitations (-1): Blinding of participants and investigators- high risk in 1 (33%) trial; Other bias- unclear risk in 3 (100%) trials. | Indirect (-1) | No important heterogeneity;  I^2^=8% | Imprecise (-1) | Very low |
| OS |  |  |  |  |  |  |  |
| Biosimilars vs. Reference biologics | 3. | 945 | Potential limitations (-1): Blinding of participants and investigators- high risk in 1 (33%) trial; Other bias- unclear risk in 3 (100%) trials. | Direct | No important heterogeneity;  I^2^=0% | Imprecise (-1) | Low |
| Incidence of grade 3-5 AEs |  |  |  |  |  |  |  |
| Biosimilars vs. Reference biologics | 3. | 945 | Potential limitations (-1): Blinding of participants and investigators- high risk in 1 (33%) trial; Other bias- unclear risk in 3 (100%) trials. | Direct | No important heterogeneity;  I^2^=0% | Imprecise (-1) | Low |

**Supplementary Table S2:** Certainty of evidence for direct, indirect and network estimates about non-small cell lung cancer patients

| **Comparisions** | **Direct estimate** | | **Indirect estimate** | | **Network estimate** | |
| --- | --- | --- | --- | --- | --- | --- |
|  | **OR/RR/HR^*^ (95%CI)** | **Quality of evidence** | **OR/RR/HR^*^ (95%CI)** | **Quality of evidence** | **OR/RR/HR^*^ (95%CI)** | **Quality of evidence** |
| **Incidence of grade 3-5 AEs** | | | | | | |
| Reference vs. PF-06439535 | 0.99 (0.65, 1.53) | High | - | - | 0.99 (0.65, 1.53) | Moderate^o^ |
| ABP 215 vs. Reference | 0.95 (0.61, 1.48) | High | - | - | 0.95 (0.61, 1.48) | Moderate^o^ |
| ABP 215 vs. PF-06439535 | - | - | 0.94 (0.5, 1.76) | High^f^ | 0.94 (0.5, 1.76) | Moderate^o^ |
| SB8 vs. ABP 215 | - | - | 1.32 (0.70, 2.46) | High^f^ | 1.32 (0.70, 2.46) | Moderate^o^ |
| SB8 vs. Reference | 1.25 (0.81, 1.93) | High | - | - | 1.25 (0.81, 1.93) | Moderate^o^ |
| SB8 vs. PF-06439535 | - | - | 1.24 (0.68, 2.28) | High^f^ | 1.24 (0.68, 2.28) | Moderate^o^ |
| FKB238 vs. SB8 | - | - | 0.74 (0.40, 1.35) | High^f^ | 0.74 (0.40, 1.35) | Moderate^o^ |
| FKB238 vs. ABP 215 | - | - | 0.97 (0.52, 1.83) | High^f^ | 0.97 (0.52, 1.83) | Moderate^o^ |
| FKB238 vs. Reference | 0.92 (0.60, 1.43) | High | - | - | 0.92 (0.60, 1.43) | Moderate^o^ |
| FKB238 vs. PF-06439535 | - | - | 0.91 (0.49, 1.69) | High^f^ | 0.91 (0.49, 1.69) | Moderate^o^ |
| MB02 vs. FKB238 | - | - | 1.19 (0.63, 2.24) | High^f^ | 1.19 (0.63, 2.24) | Moderate^o^ |
| MB02 vs. SB8 | - | - | 0.88 (0.46, 1.66) | High^f^ | 0.88 (0.46, 1.66) | Moderate^o^ |
| MB02 vs. ABP 215 | - | - | 1.15 (0.60, 2.22) | High^f^ | 1.15 (0.60, 2.22) | Moderate^o^ |
| MB02 vs. Reference | 1.09 (0.69, 1.75) | High | - | - | 1.09 (0.69, 1.75) | Moderate^o^ |
| MB02 vs. PF-06439535 | - | - | 1.09 (0.58, 2.07) | High^f^ | 1.09 (0.58, 2.07) | Moderate^o^ |
| IBI305 vs. MB02 | - | - | 1.19 (0.60, 2.41) | High^f^ | 1.19 (0.60, 2.41) | Moderate^o^ |
| IBI305 vs. FKB238 | - | - | 1.42 (0.73, 2.77) | High^f^ | 1.42 (0.73, 2.77) | Moderate^o^ |
| IBI305 vs. SB8 | - | - | 1.04 (0.54, 2.03) | High^f^ | 1.04 (0.54, 2.03) | Moderate^o^ |
| IBI305 vs. ABP 215 | - | - | 1.38 (0.70, 2.72) | High^f^ | 1.38 (0.70, 2.72) | Moderate^o^ |
| IBI305 vs. Reference | 1.30 (0.79, 2.18) | High | - | - | 1.30 (0.79, 2.18) | Moderate^o^ |
| IBI305 vs. PF-06439535 | - | - | 1.29 (0.67, 2.56) | High^f^ | 1.29 (0.67, 2.56) | Moderate^o^ |
| LY01008 vs. IBI305 | - | - | 0.58 (0.28, 1.18) | High^f^ | 0.58 (0.28, 1.18) | Moderate^o^ |
| LY01008 vs. MB02 | - | - | 0.69 (0.35, 1.38) | High^f^ | 0.69 (0.35, 1.38) | Moderate^o^ |
| LY01008 vs. FKB238 | - | - | 0.81 (0.42, 1.58) | High^f^ | 0.81 (0.42, 1.58) | Moderate^o^ |
| LY01008 vs. SB8 | - | - | 0.6 (0.31, 1.16) | High^f^ | 0.6 (0.31, 1.16) | Moderate^o^ |
| LY01008 vs. ABP 215 | - | - | 0.79 (0.41, 1.55) | High^f^ | 0.79 (0.41, 1.55) | Moderate^o^ |
| LY01008 vs. Reference | 0.75 (0.45, 1.24) | High | - | - | 0.75 (0.45, 1.24) | Moderate^o^ |
| LY01008 vs. PF-06439535 | - | - | 0.75 (0.38, 1.44) | High^f^ | 0.75 (0.38, 1.44) | Moderate^o^ |
| **ORR** | | | | | | |
| PF-06439535 vs. Reference | 1.01 (0.82, 1.25) | Moderate^c^ | - |  | 1.01 (0.82, 1.25) | Low^o^ |
| PF-06439535 vs. ABP 215 | - | - | 1.08 (0.8, 1.47) | Moderate^e^ | 1.08 (0.80, 1.47) | Low^o^ |
| PF-06439535 vs. SB8 | - | - | 0.91 (0.68, 1.22) | Moderate^e^ | 0.91 (0.68, 1.22) | Low^o^ |
| PF-06439535 vs. FKB238 | - | - | 1.05 (0.79, 1.40) | Moderate^e^ | 1.05 (0.79, 1.40) | Low^o^ |
| PF-06439535 vs. MB02 | - | - | 1.12 (0.82, 1.52) | Moderate^e^ | 1.12 (0.82, 1.52) | Low^o^ |
| PF-06439535 vs. IBI305 | - | - | 1.05 (0.76, 1.44) | Moderate^e^ | 1.05 (0.76, 1.44) | Low^o^ |
| PF-06439535 vs. LY01008 | - | - | 1.11 (0.83, 1.51) | Moderate^e^ | 1.11 (0.83, 1.51) | Low^o^ |
| Reference vs. ABP 215 | 1.07 (0.85, 1.35) | Moderate^c^ | - | - | 1.07 (0.85, 1.35) | Low^o^ |
| Reference vs. SB8 | 0.89 (0.73, 1.1) | Moderate^c^ | - | - | 0.89 (0.73, 1.1) | Low^o^ |
| Reference vs. FKB238 | 1.04 (0.86, 1.26) | Moderate^c^ | - | - | 1.04 (0.86, 1.26) | Low^o^ |
| Reference vs. MB02 | 1.11 (0.88, 1.39) | Moderate^c^ | - | - | 1.11 (0.88, 1.39) | Low^o^ |
| Reference vs. IBI305 | 1.03 (0.81, 1.32) | Moderate^c^ | - | - | 1.03 (0.81, 1.32) | Low^o^ |
| Reference vs. LY01008 | 1.1 (0.89, 1.36) | Moderate^c^ | - | - | 1.10 (0.89, 1.36) | Low^o^ |
| ABP 215 vs. SB8 | - | - | 0.84 (0.61, 1.14) | Moderate^e^ | 0.84 (0.61, 1.14) | Low^o^ |
| ABP 215 vs. FKB238 | - | - | 0.97 (0.72, 1.31) | Moderate^e^ | 0.97 (0.72, 1.31) | Low^o^ |
| ABP 215 vs. MB02 | - | - | 1.03 (0.75, 1.42) | Moderate^e^ | 1.03 (0.75, 1.42) | Low^o^ |
| ABP 215 vs. IBI305 | - | - | 0.97 (0.69, 1.35) | Moderate^e^ | 0.97 (0.69, 1.35) | Low^o^ |
| ABP 215 vs. LY01008 | - | - | 1.03 (0.75, 1.41) | Moderate^e^ | 1.03 (0.75, 1.41) | Low^o^ |
| SB8 vs. FKB238 | - | - | 1.16 (0.87, 1.53) | Moderate^e^ | 1.16 (0.87, 1.53) | Low^o^ |
| SB8 vs. MB02 | - | - | 1.23 (0.91, 1.68) | Moderate^e^ | 1.23 (0.91, 1.68) | Low^o^ |
| SB8 vs. IBI305 | - | - | 1.16 (0.84, 1.58) | Moderate^e^ | 1.16 (0.84, 1.58) | Low^o^ |
| SB8 vs. LY01008 | - | - | 1.23 (0.91, 1.66) | Moderate^e^ | 1.23 (0.91, 1.66) | Low^o^ |
| FKB238 vs. MB02 | - | - | 1.06 (0.79, 1.43) | Moderate^e^ | 1.06 (0.79, 1.43) | Low^o^ |
| FKB238 vs. IBI305 | - | - | 1.00 (0.73, 1.35) | Moderate^e^ | 1.00 (0.73, 1.35) | Low^o^ |
| FKB238 vs. LY01008 | - | - | 1.06 (0.80, 1.41) | Moderate^e^ | 1.06 (0.80, 1.41) | Low^o^ |
| MB02 vs. IBI305 | - | - | 0.94 (0.67, 1.30) | Moderate^e^ | 0.94 (0.67, 1.30) | Low^o^ |
| MB02 vs. LY01008 | - | - | 1.00 (0.73, 1.36) | Moderate^e^ | 1.00 (0.73, 1.36) | Low^o^ |
| IBI305 vs. LY01008 | - | - | 1.06 (0.77, 1.47) | Moderate^e^ | 1.06 (0.77, 1.47) | Low^o^ |
| **PFS** | | | | | | |

| PF-06439535 vs. Reference | 0.93 (0.74, 1.16) | High | - | - | 0.93 (0.74, 1.16) | Moderate^o^ |
| --- | --- | --- | --- | --- | --- | --- |
| PF-06439535 vs. SB8 | - | - | 0.94 (0.69, 1.28) | High^f^ | 0.94 (0.69, 1.28) | Moderate^o^ |
| PF-06439535 vs. FKB238 | - | - | 0.95 (0.70, 1.31) | High^f^ | 0.95 (0.70, 1.31) | Moderate^o^ |
| PF-06439535 vs. MB02 | - | - | 0.78 (0.55, 1.11) | High^f^ | 0.78 (0.55, 1.11) | Moderate^o^ |
| Reference vs. SB8 | 1.01 (0.81, 1.25) | High | - | - | 1.01 (0.81, 1.25) | Moderate^o^ |
| Reference vs. FKB238 | 1.03 (0.83, 1.28) | High | - | - | 1.03 (0.83, 1.28) | Moderate^o^ |
| Reference vs. MB02 | 0.84 (0.65, 1.10) | High | - | - | 0.84 (0.65, 1.10) | Moderate^o^ |
| SB8 vs. FKB238 | - | - | 1.02 (0.75, 1.38) | High^f^ | 1.02 (0.75, 1.38) | Moderate^o^ |
| SB8 vs. MB02 | - | - | 0.83 (0.59, 1.18) | High^f^ | 0.83 (0.59, 1.18) | Moderate^o^ |
| FKB238 vs. MB02 | - | - | 0.82 (0.58, 1.15) | High^f^ | 0.82 (0.58, 1.15) | Moderate^o^ |
| **OS** | | | | | | |
| Reference vs. PF-06439535 | 1.09 (0.87, 1.36) | High | - | - | 1.09 (0.87, 1.36) | Moderate^o^ |
| SB8 vs. Reference | 1.03 (0.84, 1.27) | High | - | - | 1.03 (0.84, 1.27) | Moderate^o^ |
| SB8 vs. PF-06439535 | - | - | 1.12 (0.83, 1.52) | High^f^ | 1.12 (0.83, 1.52) | Moderate^o^ |
| FKB238 vs. SB8 | - | - | 1.14 (0.82, 1.59) | High^f^ | 1.14 (0.82, 1.59) | Moderate^o^ |
| FKB238 vs. Reference | 1.18 (0.91, 1.52) | High | - | - | 1.18 (0.91, 1.52) | Moderate^o^ |
| FKB238 vs. PF-06439535 | - | - | 1.28 (0.92, 1.80) | High^f^ | 1.28 (0.92, 1.80) | Moderate^o^ |
| MB02 vs. FKB238 | - | - | 0.94 (0.67, 1.33) | High^f^ | 0.94 (0.67, 1.33) | Moderate^o^ |
| MB02 vs. SB8 | - | - | 1.08 (0.79, 1.47) | High^f^ | 1.08 (0.79, 1.47) | Moderate^o^ |
| MB02 vs. Reference | 1.11 (0.88, 1.40) | High | - | - | 1.11 (0.88, 1.40) | Moderate^o^ |
| MB02 vs. PF-06439535 | - | - | 1.21 (0.88, 1.66) | High^f^ | 1.21 (0.88, 1.66) | Moderate^o^ |

Note: a, limitations (Risk of bias); b, Heterogeneity; c, Indirectness; d, Publication bias; e, Contributing direct evidence of moderate quality; f, Contributing direct evidence of high quality; g, Contributing direct evidence of low quality; i, Inconsistency; o, Imprecision; q, Upgrade due to large effect; *, OR, RR, HR were adapted to evalue incidence of 3-5 grade AEs, ORR, PFS/OS respectively.

**Supplementary Table S2:** Certainty of evidence for direct, indirect and network estimates about colorectal cancer patients

| **Comparisions** | **Direct estimate** | | **Indirect estimate** | | **Network estimate** | |
| --- | --- | --- | --- | --- | --- | --- |
|  | **OR/RR/HR^*^ (95%CI)** | **Quality of evidence** | **OR/RR/HR^*^ (95%CI)** | **Quality of evidence** | **OR/RR/HR^*^ (95%CI)** | **Quality of evidence** |
| **Incidence of 3-5 grade AEs** | | | | | | |
| BEVZ92 vs. BE1040V | - | - | 0.89 (0.27, 2.98) | Moderate^e^ | 0.89 (0.27, 2.98) | Low^o^ |
| HLX04 vs. BEVZ92 | - | - | 1.07 (0.43, 2.69) | Moderate^e^ | 1.07 (0.43, 2.69) | Low^o^ |
| HLX04 vs. BE1040V | - | - | 0.96 (0.34, 2.69) | High^f^ | 0.96 (0.34, 2.69) | Moderate^o^ |
| Reference vs. HLX04 | 1.28 (0.79, 2.08) | High | - | - | 1.28 (0.79, 2.08) | Moderate^o^ |
| Reference vs. BEVZ92 | 1.37 (0.63, 3.00) | Moderate^a^ | - | - | 1.37 (0.63, 3.00) | Low^o^ |
| Reference vs. BE1040V | 1.23 (0.48, 3.05) | High | - | - | 1.23 (0.48, 3.05) | Moderate^o^ |
| **ORR** | | | | | | |
| BE1040V vs. BEVZ29 | - | - | 2.20 (0.59, 9.25) | Low^g^ | 2.20 (0.59, 9.25) | Very low^o^ |
| BE1040V vs. HLX04 | - | - | 1.98 (0.56, 8.10) | Moderate^e^ | 1.98 (0.56, 8.10) | Low^o^ |
| BE1040V vs. Reference | 1.91 (0.65, 6.65) | Moderate^c^ | - | - | 1.91 (0.65, 6.65) | Low^o^ |
| BEVZ92 vs. HLX04 | - | - | 0.90 (0.33, 2.51) | Low^g^ | 0.90 (0.33, 2.51) | Very low^o^ |
| BEVZ92 vs. Reference | 0.87 (0.42, 1.83) | Low^a,c^ | - | - | 0.87 (0.42, 1.83) | Very low^o^ |
| HLX04 vs. Reference | 0.96 (0.48, 1.93) | Moderate^c^ | - | - | 0.96 (0.48, 1.93) | Low^o^ |
| **OS** | | | | | | |
| BE1040V vs. HLX04 | - | - | 1.08 (0.92, 1.27) | High^f^ | 1.08 (0.92, 1.27) | Moderate^o^ |
| BE1040V vs. Reference | 1.00 (0.90, 1.10) | High | - | - | 1.00 (0.90, 1.10) | Moderate^o^ |
| HLX04 vs. Reference | 0.92 (0.81, 1.04) | High | - | - | 0.92 (0.81, 1.04) | Moderate^o^ |

Note: a, limitations (Risk of bias); b, Heterogeneity; c, Indirectness; d, Publication bias; e, Contributing direct evidence of moderate quality; f, Contributing direct evidence of high quality; g, Contributing direct evidence of low quality; i, Inconsistency; o, Imprecision; q, Upgrade due to large effect; *, OR, RR, HR were adapted to evalue incidence of 3-5 grade AEs, ORR, OS respectively; CI, confidence interval; OR, odds ratio; HR, hazard ratio; RR, risk ratio; ORR: Objective response rate; PFS: Progression-free survival; OS: Overall survival; AEs: adverse events.


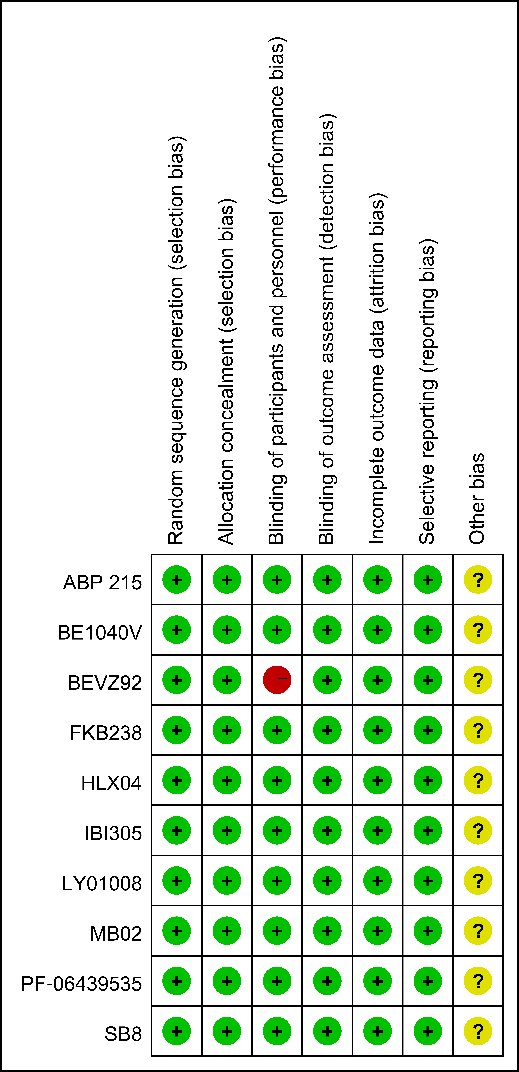


**Supplementary Figure S1:** Risk of bias assessment. Green was for low risk, yellow was for unclear, and red was for high risk.

**
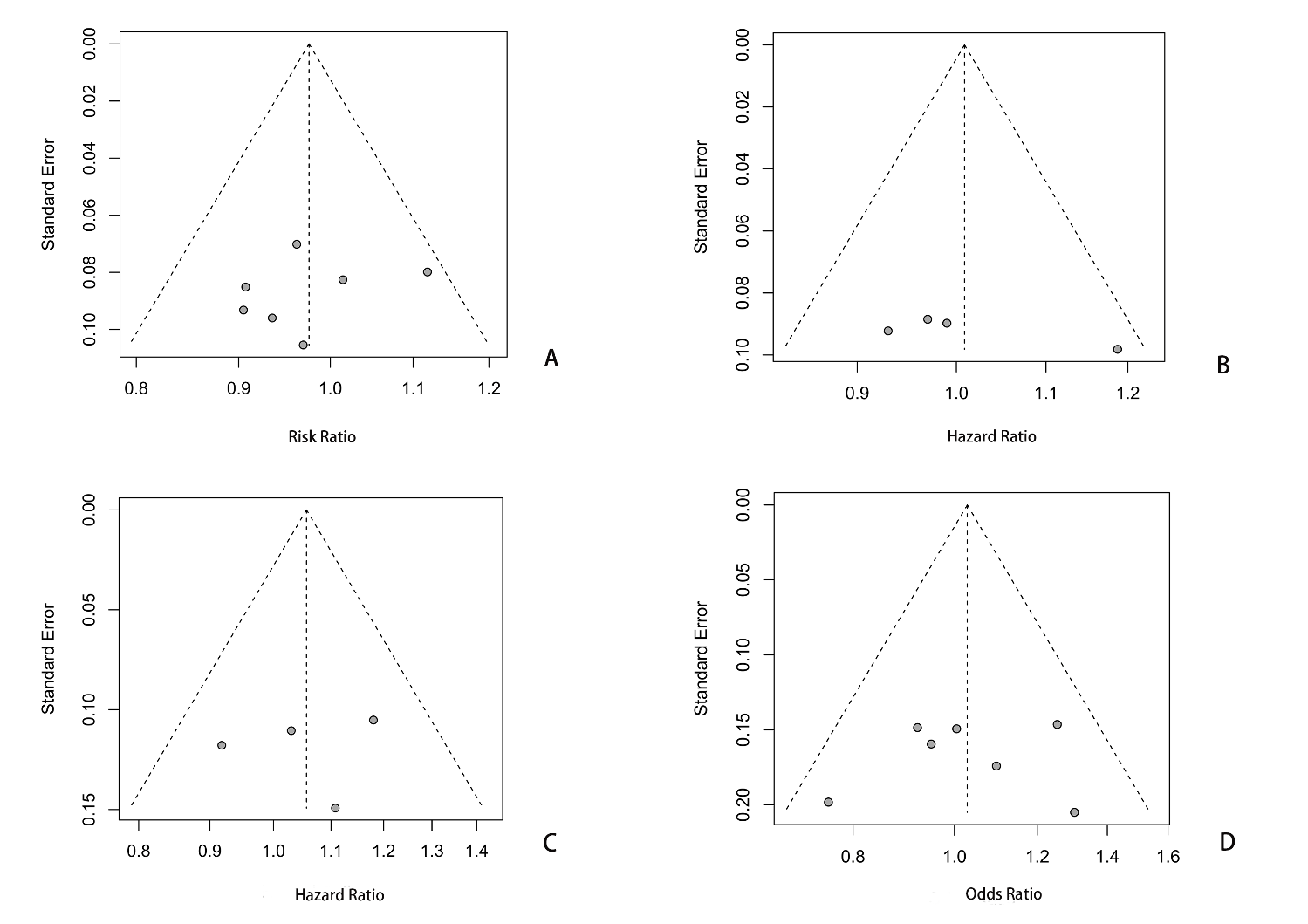
**

**Supplementary Figure S2:** Funnel plot for non-small cell lung cancer patients

**(A)** Objective response rate; **(B)** Progression-free survival; **(C)** Overall survival; **(D)** Incidence of grade 3-5 adverse events


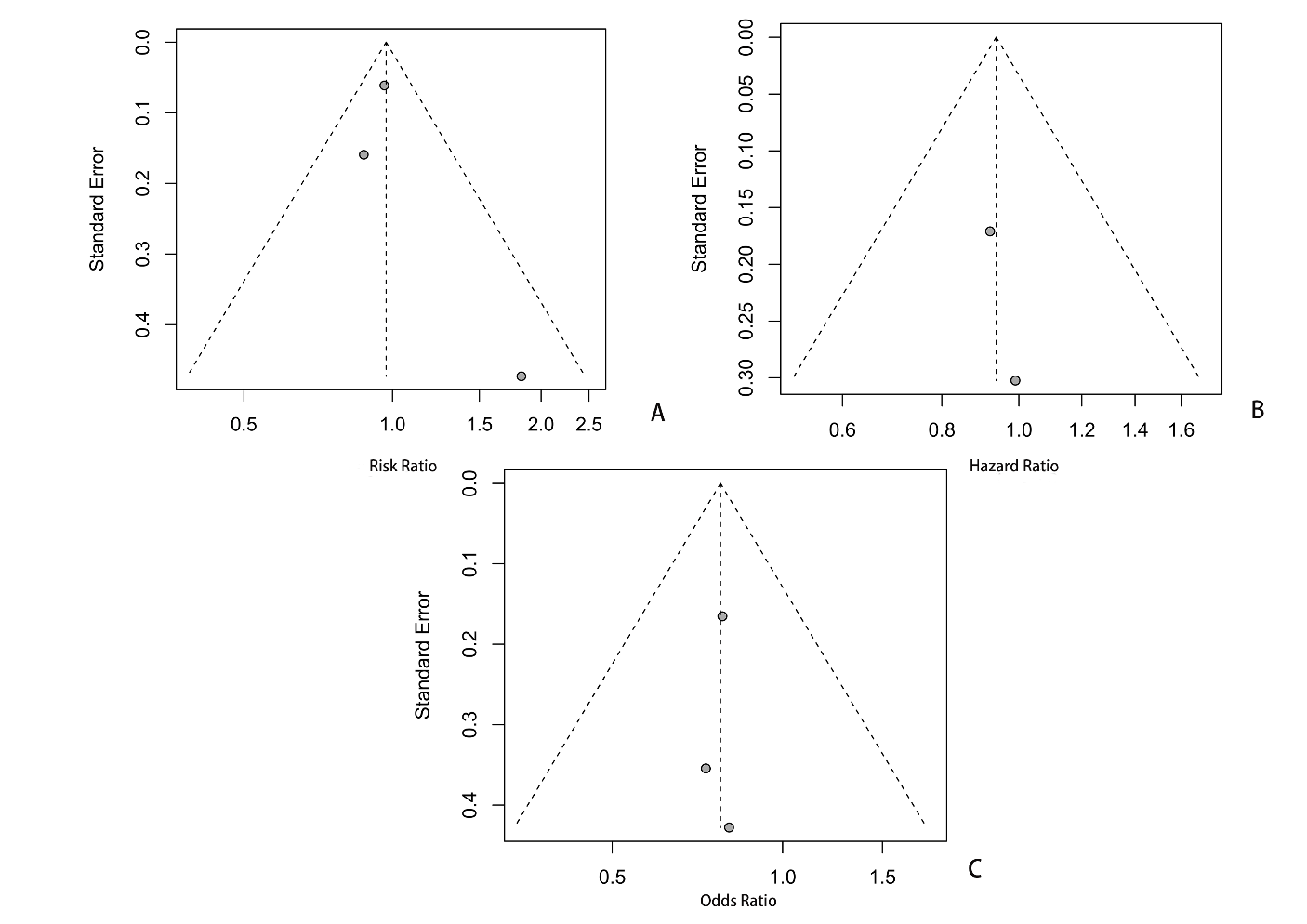


**Supplementary Figure S3:** Funnel plot for colorectal cancer patients

**(A)** Objective response rate; **(B)** Overall survival; **(C)** Incidence rate of grade 3-5 adverse events
